# Biochemical and molecular characterization of novel keratinolytic protease from *Bacillus licheniformis* (KRLr1)

**DOI:** 10.3389/fmicb.2023.1132760

**Published:** 2023-05-10

**Authors:** Somayyeh Rahimnahal, Amir Meimandipour, Jamal Fayazi, Ali Asghar Karkhane, Mehdi Shamsara, Mohammadtaghi Beigi Nassiri, Hamed Mirzaei, Michael R. Hamblin, Hossein Tarrahimofrad, Hamid Bakherad, Javad Zamani, Yahya Mohammadi

**Affiliations:** ^1^Department of Animal Science and Food Technology, Agriculture Science and Natural Resources University Khouzestan, Ahwaz, Iran; ^2^Department of Animal Biotechnology, National Institute of Genetic Engineering and Biotechnology, (NIGEB), Tehran, Iran; ^3^Research Center for Biochemistry and Nutrition in Metabolic Diseases, Institute for Basic Sciences, Kashan University of Medical Sciences, Kashan, Iran; ^4^Faculty of Health Science, Laser Research Centre, University of Johannesburg, Johannesburg, South Africa; ^5^Department of Pharmaceutical Biotechnology and Isfahan Pharmaceutical Sciences Research Center, School of Pharmacy and Pharmaceutical Sciences, Isfahan University of Medical Sciences, Isfahan, Iran; ^6^Department of Animal Science, Ilam University, Ilam, Iran

**Keywords:** HPLC, thermodynamics, cloning, keratinase, molecular dynamics

## Abstract

The keratin-degrading bacterium *Bacillus licheniformis* secretes a keratinase with potential industrial interest. Here, the Keratinase gene was intracellularly expressed in *Escherichia coli* BL21(DE3) using pET-21b (+) vector. Phylogenetic tree analysis showed that KRLr1 is closely related to *Bacillus licheniformis* keratinase that belongs to the serine peptidase/subtilisin-like S8 family. Recombinant keratinase appeared on the SDS-PAGE gel with a band of about 38 kDa and was confirmed by western blotting. Expressed KRLr1 was purified by Ni-NTA affinity chromatography with a yield of 85.96% and then refolded. It was found that this enzyme has optimum activity at pH 6 and 37°C. PMSF inhibited the KRLr1 activity and Ca^2+^ and Mg^2+^ increased the KRLr1 activity. Using keratin 1% as the substrate, the thermodynamic values were determined as K_m_ 14.54 mM, k_cat_ 912.7 × 10^−3^ (S^−1^), and k_cat_/K_m_ 62.77 (M^−1^ S^−1^). Feather digestion by recombinant enzyme using HPLC method, showed that the amino acids cysteine, phenylalanine, tyrosine and lysine had the highest amount compared to other amino acids obtained from digestion. Molecular dynamics (MD) simulation of HADDOCK docking results exhibited that KRLr1 enzyme was able to interact strongly with chicken feather keratine 4 (FK4) compared to chicken feather keratine 12 (FK12). These properties make keratinase KRLr1 a potential candidate for various biotechnological applications.

## Introduction

It has been realized that enzymes are a major factor in green processing industries, which are important for enhancing the quality of life (Nnolim et al., 2020). Enzymes are proteins that act as catalysts to accelerate a specific chemical reaction. Enzyme biotechnology can be used to create useful products, such as biofuels, or to purify and process industrial materials. It can also be used to break down complex materials into simpler components, such as breaking down proteins into amino acids. Keratin is the main structural component of many animal tissues that are made of keratin, including feathers, wool, claws, and so on. Keratin is an insoluble macromolecule with high stability and low decomposition rate and is present in hair, feathers, nails, wool, beak, and horn (Onifade et al., 1998). Keratin is divided into two groups: α-keratin (hair, nails, horns, etc.) and β-keratin (feathers, scales, beak, paw, etc.) (Mazotto et al., 2011). The high protein content of keratin waste can be converted into a good source of protein and amino acids using the recycling cycle (Adler et al., 2018). Microbial decomposition of feathers is considered by most scientists and researchers to be a suitable alternative to alkaline and vapor hydrolysis, which is considered in terms of preserving essential nutrients and amino acids. Keratinase is the only group of proteases with a wide range of temperature and pH that can destroy complex and reversible proteins such as casein, collagen, elastin, and keratin and other proteins containing cysteine disulfide bonds (Gupta and Ramnani, 2006). A unique feature that distinguishes keratinases from other proteases is the ability to bind to complex and insoluble substrates (feathers, wool, silk, collagen, elastin, horns, hair, azokeratin, and nails) (Gupta and Ramnani, 2006). The extent and abundance of disulfide and hydrogen bonds give keratin its remarkable resistance and imperviousness to common chemicals and proteolytic enzymes like pepsin and trypsin (Zhang et al., 2022). Microbial keratinase is able to break down the difficult to process keratin protein into smaller peptides or amino acids. Microbial keratinase’s impressive ability has caused many researchers to take a keen interest in it. Keratinase is seen as a valuable industrial enzyme due to its ability to target keratin-based substrates, its strong activity at a high pH, and its applications in areas such as detergent production, leather dehairing, feed production, and waste handling (Williams, 1991; Ben Elhoul et al., 2016; Srivastava et al., 2020). Reports has been shown that the vast amount of chicken feathers produced annually and how they are recycled for various uses. Initially, decomposition of alkaline hydrolysis and vapor pressure has been used, but these methods were expensive and the result was low in terms of nutrients. Therefore, in recent years, to overcome this limitation, the use of microbial enzymes to improve nutritional value has become widespread (Kumar et al., 2012). Keratinase has been used to improve feather digestibility for use in the diets of livestock and poultry (Kim and Patterson, 2000). Keratinase supplements in corn-soy-based diets have also been shown to improve intestinal function and amino acid intake (Odetallah et al., 2003, 2005; Wang et al., 2006, 2008).

A large number of studies have shown the high capability of keratinase enzymes of *Bacillus* origin (Laurila, 2013; Hamiche et al., 2019; Jana et al., 2022; Sharma et al., 2022; Yahaya et al., 2022; Zhang et al., 2022). However, obtaining keratinase in high amounts requires the use of recombinant protein heterologous expression systems (Ben Elhoul et al., 2021). Therefore, we used *E. coli* to obtain recombinant keratinase.

The aim of this study was to the expression of the keratinase gene derived from *Bacillus licheniformis* KRLr1 from poultry litter for feather digestion to release its amino acids for use in livestock and poultry feed. Recombinant keratinase was expressed and purified and evaluated for biophysical and biochemical properties such as molecular docking and molecular dynamics simulation, optimum temperature and pH, and kinetic parameters. Finally, the chicken feathers were subjected to the digestion of the recombinant enzyme and the resulting amino acids were identified by HPLC.

## Materials and methods

### Chemicals, bacterial strains, and culture conditions

Taq DNA polymerase, all enzymes, DNA and protein ladder were purchased from Fermantase (United States). The DNA extraction kit was purchased from Peqlab and the PCR extraction kit, plasmid and Master Mix PCR kit were purchased from Bioneer (Korea). Penta • His Antibody, 100 μg mouse anti- (His) 5 (Cat. No. 34660) and HRP-Poly Histidin (goat anti-mouse IgG—HRP-conjugate from Jackson Immunoresearch (Cat. No. 115–035 -003) was purchased from Qiagen Company. ECL (Cat. No. RPN2235) detection kit was purchased from Amersham. Ni-NTA resin was purchased from Invitrogen (Carlsbad, United States). The *E. coli* DH5α and *E. coli* BL21 strains were purchased from Invitrogen (Carlsbad, United States). The LB general culture medium was used for bacterial strain culture. The keratin substrate for the culture medium was prepared according to Wawrzkiewicz et al. (1987) and Riffel et al. (2003). All other chemical materials and culture medium were obtained from Merck Company. *E. coli* strain containing pET-21b (+) vector was cultured on agar LB culture medium containing 100 μg/mL ampicillin at 37°C.

### Structural studies

#### *In silico* and phylogenetic analysis of keratinase

The nucleotide sequence of the keratinase gene after sequencing was translated into the amino acid sequence using the Expasy translate tool.^1^ The biochemical properties of the protein were calculated using the Expasy ProtParam tool.^2^ The keratinase amino acids sequence was investigated for the presence of the peptide signal and the disulfide bond using the SignalP 4.1 and DiANNA 1.1 servers, respectively. The GC percentage of the enzyme encoding sequence was calculated using the Genom GC Calculator.^3^ Sequence alignment and the identification of the peptidase family were performed using databases pFam^4^ and MEROPS.^5^ Using DNAMAN software, multiple alignments of enzyme amino acid sequences were conducted. Phylogenetic relationships of the amino acids sequence were analyzed using ClustalW in MEGA 8 software.

#### Homology modeling and validation

The secondary protein structure was predicted using the NetSurfP-2.0 program.^6^ The MODELLER 9V7 program was used to predict the third structure of the protein. Briefly, PDB-Blast amino acid sequence of keratinase was performed in NCBI. Then, the crystallographic PDB structure of Chain A, Subtilisin (*Bacillus subtilis*) with 3WH RCSB code of the keratinase family, which had the most similarity and e-value with KRLr1 enzyme, was introduced to MODELLER 9 V7 software.

The refining of the 3D modeled keratinase was performed by the ModRefiner server. The Ramachandran plot graph was drawn by the online website.^7^ For ensuring the accuracy of the simulated building the http://services.mbi.ucla.edu/PROCHECK and http://services.mbi.ucla.edu/SAVES were used. The website https://prosa.services.came.sbg.ac.at/prosa.pHp was used to determine the Z-Score point and protein energy level. With Chimera v1.13.1 software, the best spatial position for the optimal energy of the keratinase enzyme was determined. Using COFACTOR^8^ the active site was predicted of the proposed model and visualized using the Chimera V1.13.1 program.^9^

Also, the third structure relative to feather keratin 4 (FK4) and feather keratin 12 (FK12) of chicken that is located on chromosome 2 and chromosome 25 were retrieved from NCBI (gi|971385219 and gi|480540358 accession number, respectively) and were submitted to I-TASSER server in https://zhanglab.ccmb.med.umich.edu/I-TASSER/ for predicting of its 3D structure. Furthermore, cleavage sites on FK4 and FK12 amino acids were determined in the PeptideCutter tool in the Expasy server^10^ with assuming of serine peptidase as a KRLr1 keratinolytic template activity on keratin substrate.

#### Protein–protein docking studies and analysis

The KRLr1, FK4, and FK12 predicted structures were prepared for docking in the Chimera V1.131, so that:

I) All three KRLr1, FK4, and FK12 structures were introduced to Chimera V1.131, separately and all of the water molecules were removed from around PDBs.
II) Then all three KRLr1, FK4, and FK12 structures were “energy minimized” in the Chimera V1.131. In this step, all three KRLr1, FK4, and FK12 structures were saved as PDB format.
III) The PDB file of KRLr1 structure was called as “First molecule” in HADDOCK server.^11^
IV) Then the amino acids of its active site were selected and introduced to “Active residues (directly involved in the interaction)” in HADDOCK server, based on the information obtained from the 3D Ligand server.
V) The PDB file of FK4 structure was called as “Second molecule.”
VI) Then the amino acids of its calvage site were selected and introduced to “Active residues (directly involved in the interaction)” in HADDOCK server, based on the information obtained from the PeptideCutter server.
VII) Then, the complex (KRLr1-FK4) was submitted and was run as Protein/Protein docking.
VIII) Once again, all the above steps were done separately for KRLr1-FK12 complex.

Finally, the docked complex (separately for each one of the KRLr1-FK4 and KRLr1-FK12 complexes) with lower binding energy from the HADDOCK scores best cluster was selected for MD simulation.

#### Molecular dynamics simulation

The KRLr1-FK4 and KRLr1-FK12 complexes were simulated for 50 ns time using GROMACS v.5.1.4 and by following the protocol in http://manual.gromacs.org/documentation/5.1.4/index.html. The trajectories were saved for each complex after every 2 fs and the CGenFF server^12^ provides topologies and parameters of ligands compatible with the CHARMM36 all-atoms force field. The protein–protein complexes were filled in a cubic box of water molecules, and the proteins were neutralized by the addition of Na^+^ and Cl^−^ charging ions. The steepest descent algorithm was used for energy system minimization and using the LINCS algorithm, all bonds were constrained. The desired temperature (300 K protein–protein complexes) and pressure (1 bar) for 100 ps and restraint forces of 1,000 kJ/mol were achieve using The NVT and NPT ensembles during the equilibration. Eventually, the gmx rmsd, gmx rmsf, gmx gyr, and gmx hydrogen bond module of GROMACS were used to analysis of root-mean-square deviation (RMSD), root-mean-square fluctuations (RMSF), the radius of gyration (Rg) and hydrogen bond analysis, respectively.

#### MMP/BSA calculations of MD results

The Molecular Mechanics Poisson–Boltzmann Surface Area (MMP/BSA) method implemented in AMBER 14 was used to calculate the binding free energies. The 100 snapshots of the equilibrium stage were used from the MD trajectory for each system and free energy was calculated for KRLr1-FK4 and KRLr1-FK12 complexes using a 50 ns simulation trajectory. The total binding free energy of protein with protein insolvent was calculated using the following equation:


(1) $$\Delta G_{\mathrm{bind}}=\Delta G_{\mathrm{complex}}-\Delta G_{\mathrm{receptor}}-\Delta G_{\mathrm{protein}}$$

(2) $$\Delta G_{\mathrm{bind}}=\Delta E_{\mathrm{MM}}+\Delta G_{\mathrm{solv}}-T\Delta S$$

(3) $$\Delta E_{\mathrm{MM}}=\Delta E_{\mathrm{vdw}}+\Delta E_{\mathrm{ele}}$$

(4) $$\Delta G_{\mathrm{solv}}=\Delta G_{\mathrm{PB}}+\Delta G_{\mathrm{SA}}$$

here ΔE_MM_ exhibits the gas-phase interaction energy between the receptor and the protein which including van der Waals energy contribution (ΔE_vdw_) and electrostatic energy contribution (ΔE_ele_); ΔGPB and ΔGSA are the polar and nonpolar components of the de-solvation free energy, respectively; TΔS represents the conformational entropy contribution at temperature T.

#### Cell culture and keratinase gene amplification

Three milliliter [5.4 × 10^9^ cells/mL in the culture medium with a turbidity of 0.4 (OD) at a wavelength of 600 nm] of the culture medium, harvested and were centrifuged at 4,000 × g, and 4°C for 10 min. Genomic DNA was extracted using a DNA purification kit according to the manufacturer’s instructions.

The keratinase gene was isolated by PCR with the specific primers kerF:(5′-GCATATGTGAGGAAAAAGAGTTTTTG-3′) and kerR:(5′-ATCTCGAGTTGAGCGGCACCTTCGAC-3′) with underlined NdeI and XhoI restriction enzyme sites, respectively. Both forward and reverse primers were designed according to the nucleotide sequence of the *Bacillus licheniformis* MKU3 (DQ071570.1).

#### Codon optimization and heterologous expression of keratinase gene

The sequence of the keratinase gene was investigated for the presence of rare codons using the online GenScript program. According to the results of the study of rare codons, parts of this sequence were identified and sent to the Media Economics Company, Tehran, for optimization. The keratinase gene was synthesized in the pET-21b(+) expression vector. The recombinant pET-21b(+)-ker was transferred into *E. coli* DH5α by thermal shock method, then the recombinant vector was transferred into *E. coli* BL21 (DE3). Transformed bacteria were cultured on agar LB medium containing ampicillin (100 μg/mL). The transformed bacteria were cultured on the LB agar medium containing ampicillin (100 micrograms per milliliter) and incubated overnight at 37°C. A positive BL21 bacterial colony (containing a recombinant expression vector) was incubated overnight at 37°C (180 rpm) in 5 mL LB medium containing ampicillin (100 μg/mL) and inoculated to the fresh medium (1/50 mL) with the same antibiotic concentration.

#### Recombinant protein purification

##### Recovery of recombinant keratinase from inclusion bodies

The modified Qiagen handbook for protein purification under denaturing conditions was used to make the protein solution. After preparation of the expression sample, 50 mL of the culture medium containing bacteria were centrifuged at 8,000 g at 4°C for 20 min. After discarding the supernatant, the bacterial precipitate was resuspended in a 5 mL lysis buffer (100 mM NaH_2_PO_4_, 10 mM Tris–HCL, and 6 mM GuHCL at a pH of 7.8). The mixture was sonicated with 3 × 90 s pulses followed by 30 s rest between cycles at 4°C. In this method, the sonic solution was placed at room temperature on a shaker at a very low speed for 2 h without centrifugation (the speed of the shaker should be low enough to prevent foam formation). Then, the lysed solution was divided into 1.5 mL micro-tubes and centrifugation was performed with the maximum possible speed (13,000 rpm) at 4°C for 40 min.

##### Ni-NTA affinity chromatography

In brief, 1 mL chromatography column of Ni-NTA resin was prepared. To balance the resin, the column was first washed with a 2–4 mL Denaturing Binding Buffer (100 mM NaH_2_PO_4_, 10 mM Tris–HCL, and 8 M Urea at pH 8). Then, 4 mL of the protein solution from the sonication was transferred to the column and placed at room temperature on the shaker for 2 h at a very slow speed (the resin in the column at this stage was completely dissolved in the solution from the sonicate). After the above time, the column was placed in its place and after complete resin settling, the protein soup was passed. Then, the column was washed three times with a buffer (100 mM NaH_2_PO_4_, 10 mM Tris–HCL, 8 M Urea, and 10 mM Imidazole at pH = 8), three times with an elution buffer (100 mM NaH_2_PO_4_, 10 mM Tris–HCL, 8 M Urea, and 20 mM Imidazole at pH = 8) and four times with an elution buffer containing 40 and 250 mM imidazole, respectively (pH = 8). All the collected fractions were visualized using 12% sodium dodecyl sulfate-polyacrylamide gel electrophoresis.

##### Refolding of solubilized protein

To remove urea and imidazole and then refold the 3D structure of the purified enzyme by the Ni-NTA chromatography, dialysis was performed using a suitable cofactor. After preparing the dialysis bag (5 × 5 cm^2^), 5 mL of the purified enzyme was added to it and the lid of the bag was tightly closed with a special clamp. The enzyme-containing dialysis bag was then placed in a 500 mL potassium phosphate buffer containing 6 M, 4 M, 2 M of urea and no urea, each containing 0.5 mM molybdenum ions (MOO3) cofactor. Each buffer was used for 6 h on a magnet stirrer at 4°C.

#### Western blotting

After transferring the proteins from the gel to the nitrocellulose membrane, the membrane was blocked at room temperature for 1 h with TBST (25 mM Tris–HCl, pH 7.4, 0.14 mM NaCl, and 0.05% Tween 20) containing 5% BSA. The membrane was washed three times with TBST and incubated overnight in a 1:20,000 diluted Penta•His antibody at 4°C. After three more washes with the TBST, the HRP-Poly Histidin conjugated antibody was prepared at a concentration of 1:200 and added to the membrane for 2 h in the dark at 4°C. Next, the nitrocellulose membrane was removed from the secondary antibody solution and immersed twice in TBST buffer and then the blot was washed at 4 mL/cm^2^ of the wash solution for 15 min at room temperature on a shaker. The wash was repeated three more times each time for 5 min in fresh buffer. An ECL Kit was used to detect the protein.

#### Keratinase activity assay

The keratinase activity was determined by the method of Lin and Yin (2010) using 1% keratin in 50 mM potassium phosphate buffer (pH 7.5) as a substrate. For this purpose, 250 μL of the purified enzyme was dissolved in 750 μL of the substrate and the control sample containing these compounds plus 1 mL of 15% TCA was added and incubated for 60 min at 37°C at 180 rpm. After this time, 1 mL of fresh and cool 15% TCA was added to the sample vial. Both sample and control vials were placed in ice for 30 min at the same time. The vials were centrifuged at 10,000 g for 10 min. The absorbance of the supernatant was measured at 280 nm. This experiment was repeated three times.

#### Effects of temperature and pH on the keratinase activity and stability

To investigate the effect of temperature on enzyme activity, the reaction of enzyme activity was performed according to the modified method of Lin and Yin (2010) at temperatures of 15, 20, 30, 37, 40, 50, 60, 70, and 80°C for 90 min and with 30 min intervals at pH 6–7.

To determine the pH profile of the enzyme, the keratinase activity was performed in a pH range of 3–10 using 50 mM acetate (pH 3–5.6), 50 mM potassium phosphate (pH 6–7), 50 mM Tris–HCL (pH 8–9) and 50 mM Glycine-NaOH (10 pH) buffers were used to determine the pH profile of the enzyme activity (Manonmani et al., 2015). Enzyme activity assays were performed at different pHs at the optimum temperature (37°C). Each experiment was performed three times and the enzyme activity diagram was drawn at different temperatures and pHs using Prism 6 software.

#### Effect of metal ions, inhibitors, organic solvents, on keratinolytic activity

The KRLr1 activity was examined with various metalic ions, such as 5 and 10 mM concentrations of Mg^2+^, Hg^2+^, Cu^2+^, Co^2+^, Zn^2+^, Fe^2+^, K^+^, Ca^2+^, Mn^2+^, Ba^2+^, LiSo_4_, and Na^+^. The potential impact of enzyme inhibitors was assessed in 1 and 5 mM concentrations of various chemicals, including guanidine hydrochloride (GuHCl), phenyl-methylsulfonyl fluoride (PMSF), iodoacetamide (IAA), E-64 [Trans-Epoxysuccinyl-L-leucylamido (4-guanidino) butane], ethylenediaminetetraacetic acid (EDTA), Leupeptin, β-mercaptoethanol (2-ME), and urea. Then we explored influence of surfactants and organic solvents, including SDS, Tween-20, isopropanol, methanol, SDS, isobutanol and glycerol (at volumes of 10 and 20% respectively) on enzyme activity. The KRLr1 activity was assumed to be the control (100% activity), without any additional substances in the assay reaction solution.

#### Thermodynamic study

Arrhenius equation was used for the calculation of KRLr1 activation energy (E_a_^‡^), so that:


(2) $$\alpha =-E_{a}{}^{{}^{‡}}/R$$

α is the slope obtained from the calculation of 1/T vs. Ln [k_a_], and R is the gas constant (8.314 J/K mol).


(3) $$k_{\mathrm{cat}}=\left(k_{B}\hbar \right)\times k_{\mathrm{cat}}{}^{{}^{‡}}$$

k_B_ and ℏ are the Boltzmann and Planck constants, respectively, and N is the Avogadro’s number.


(4) $$\Delta G^{{}^{‡}}=-\mathrm{RT}\;\left[\mathrm{Ln}\;k_{\mathrm{cat}}{}^{{}^{‡}}\right]$$

T is the temperature in Kelvin.

The ∆G^‡^ is the changes in the Gibbs free energy of activation energy.


(5) $$\Delta H^{‡}=E_{a}{}^{‡}\mathrm{-RT}$$

The ∆H^‡^ is the changes in the enthalpy energy,


(6) $$\Delta S^{{}^{‡}}=\Delta H^{{}^{‡}}-\Delta G^{{}^{‡}}/T$$

The ∆S^‡^ is the changes in entropy energy.

[ΔG^‡^_Enzyme-Substrate_] is calculated as:


(7) $$\Delta G^{{}^{‡}}{}_{\mathrm{Enzyme}-\mathrm{Substrate}}=-\mathrm{RT}\;\mathrm{Ln}\;K_{i}\;\mathrm{that}\;K_{i}=1/K_{m}$$

Free energy of the transition state (TS) [ΔG^‡^_Enzyme-Substrate_] formation is obtained as:


(8) $$\Delta G^{{}^{‡}}{}_{\mathrm{Enzyme}-\mathrm{Transition state}}=-\mathrm{RT}\;\mathrm{Ln}\;\left(k_{\mathrm{cat}}/K_{m}\right)$$

KRLr1 temperature stability graph was used for calculation of irreversible inactivation, so the inactivation constant (k_in_ m^−1^) was calculated as:


(9) $$\mathrm{Ln}\;\left(\left[\mathrm{Act}\right]t/\left[\mathrm{Act}\right]0\right)=-k_{\mathrm{in}}t$$

t is the enzyme incubation time, [Act]0 is the KRLr1 activity at time 0, and [Act]t is the activity at the time “t” of reaction.

The KRLr1 half-life (t_1/2_) was measured as:


(10) $$t_{1/2}=\ln2/k_{\mathrm{in}}{\left(\min\right)}^{-1}$$

This goes on to calculate E_a_^#^ based on the equation of Arrhenius:


(11) $$k_{\mathrm{in}}=\mathrm{Ae}\left({\mathrm{-E}}_{a}{}^{\#}/\mathrm{RT}\right)$$

such that.

(12) $$\mathrm{Ln}\left[k_{\mathrm{in}}\right]=-E_{a}{}^{\#}/\mathrm{RT}$$

The values of ΔG^#^, ΔH^#^, and ΔS^#^ related to KRLr1 were determined through applying Eqs.

#### Kinetic parameters analysis and calculation

GraphPad Prism V.8 was used to perform non-linear regression to characterize the kinetic properetises (K_m_, V_max_, k_cat_, and k_cat_/K_m_) of the pure KRLr1 in terms of Michaelis/Menten kinetic parameters (Mohammadi et al., 2022). A variety of keratin substrate concentrations (10–70 mM) and the activity optimum temperature were used to measure the keratinase activity. Assays were carried out in triplicate and with 0.6 mg/mL of the purified KRLr1 (Shahi et al., 2021).

#### Feather digestion using recombinant enzymes and analysis of amino acids

To investigate the digestion of the feather using the recombinant enzyme, 2 mL of the enzyme was added to 100 mL of feather broth (FB) medium containing 1% chopped feather (1–2 cm pieces). The inoculated medium was incubated at 37°C at 180 rpm for complete digestion. After the incubation period, first the culture medium was centrifuged at 8,000 rpm for 8 min and the supernatant was separated and the same volume of TCA was added, then it was incubated for 30 min at 4°C. The resulting solution was centrifuged again and then the supernatant was filtered with a 0.2 μm membrane filter. Soluble amino acid compounds were determined by HPLC with o-phthalaldehyde-9-flourenylmethyl chloroformate precolumn derivatization (Rajagopal et al., 2016). The VWD detector was used, the wavelength was 338 nm and the flow rate was 1 mL/min. The column was Hypersil ods-2 with a temperature of 40°C and an injection volume of 10 microliters. Soluble peptides were determined by comparing peak time and peak location.

## Results

**Figure 1:**
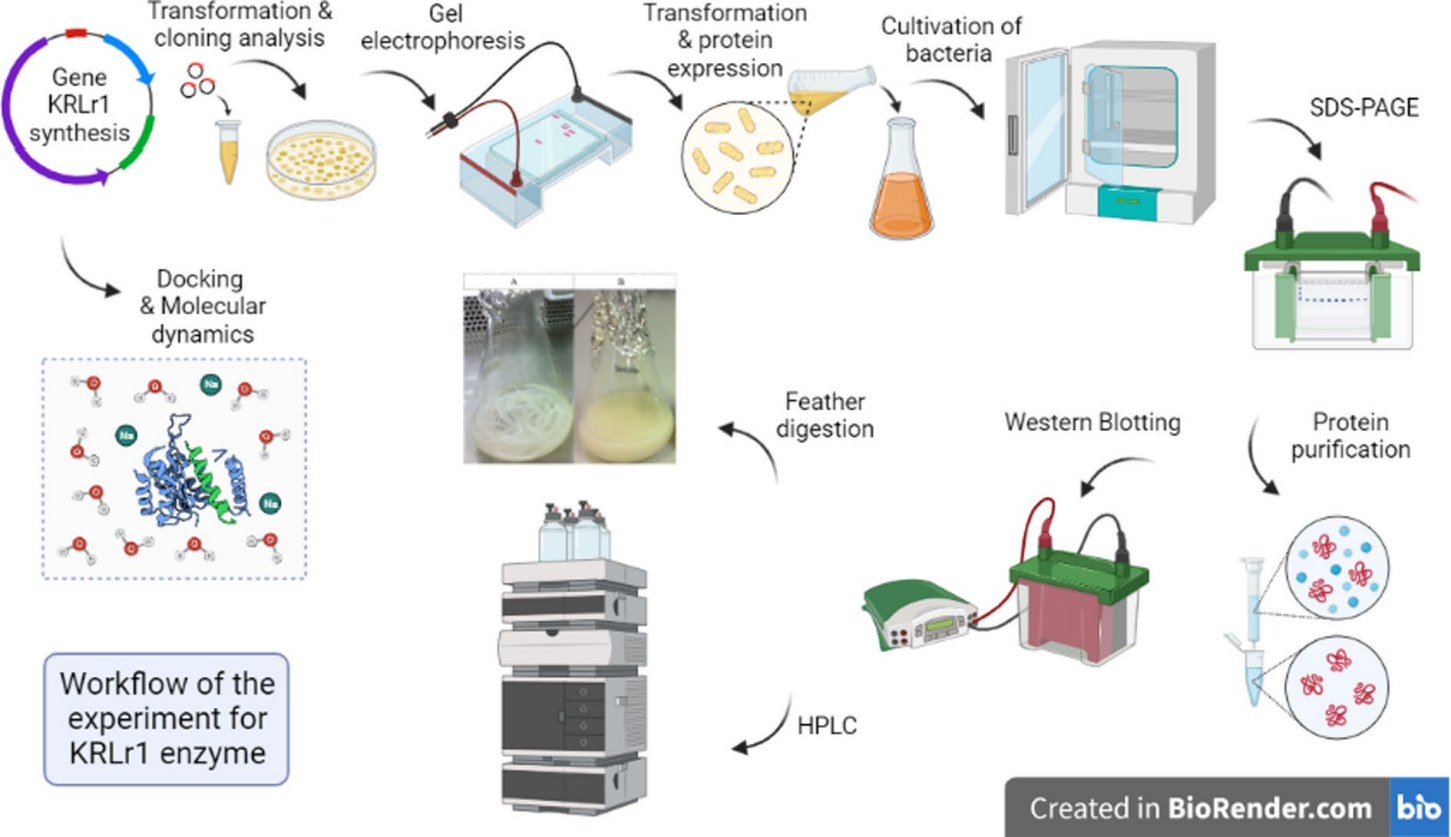
Workflow of the experiment on the flow chart model.

All the steps of this study are shown as a workflow (Figure 1).

### Keratinase gene amplification and expression of the keratinase enzyme

**Figure 2:**
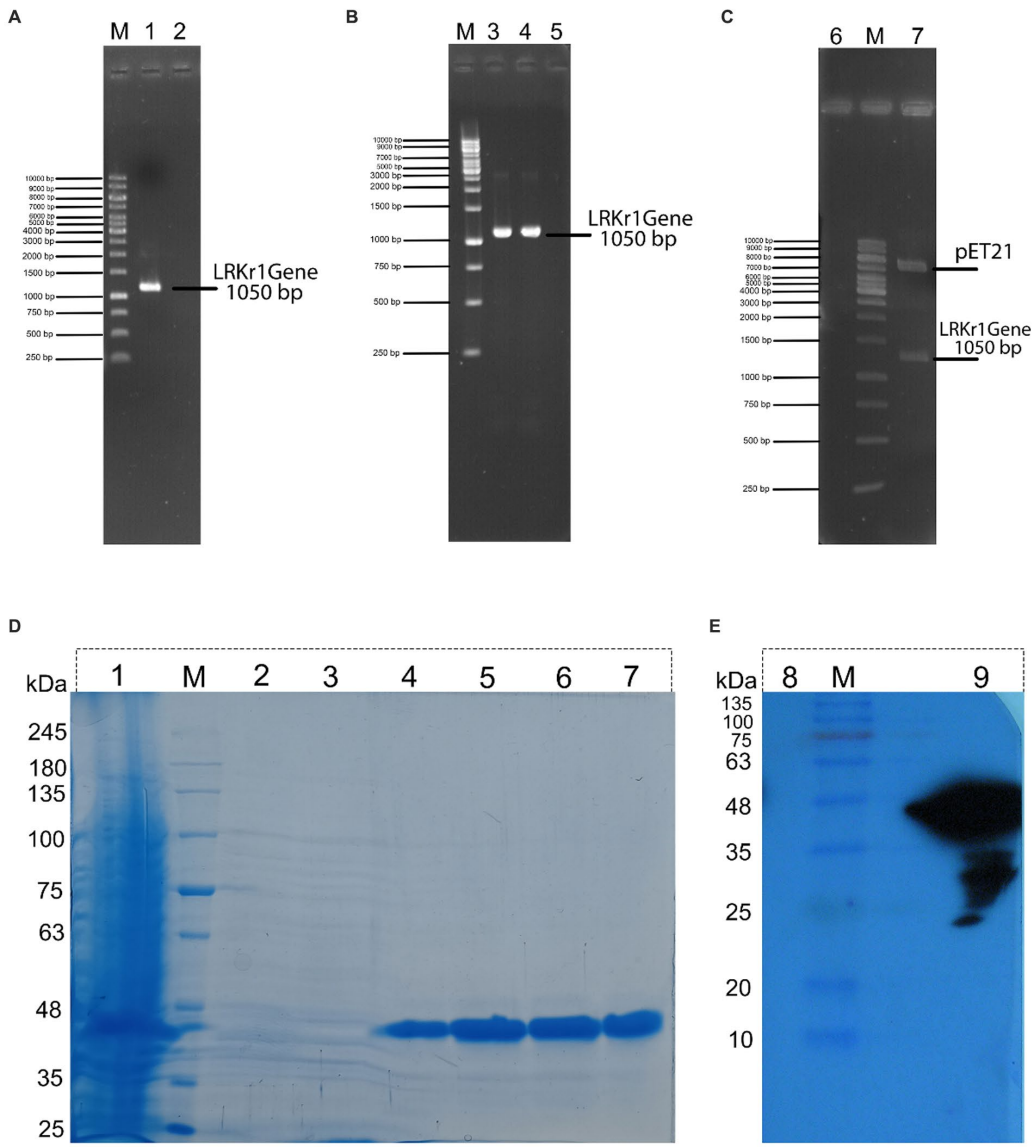
**(A)** (M) DNA marker; (1) PCR from genomic DNA of keratinase sequence; (2) negative control. **(B)** (M) DNA marker; (3) PCR from recombinant BL21 contain recombinant pET21 (colony PCR); (4) PCR from recombinant pET21 vector with specific primers for keratinase sequence; (5) negative control. **(C)** (6) negative control; (M) DNA marker; (7) double digestion of recombinant pET21 by XhoI and NdeI restriction enzyme. **(D)** (1) expression of 16 h of bacteria, (M) Molecular marker of protein; (2) protein passing through the column with buffer containing 10 mM imidazole; (3) washing the column with buffer containing 20 mM imidazole; (4) washing the column with buffer containing 120 mM of imidazole; (5) wash the column with a buffer containing 250 mM of imidazole; (6) wash the column with a buffer containing 250 mM of imidazole (repeat run); (7) wash the column with a buffer containing 500 mM of imidazole. **(E)** Western blot of recombinant protein. (8) negative control (BSA); (M) Molecular marker of protein; and (9) keratinase with a molecular weight of about 38.5 kDa.

After amplification of the keratinase gene using the PCR method, the product was analyzed on 1% agarose gel. As shown in Figure 1A, the keratinase gene was well amplified at 1,050 bp. The amplified gene was sequenced after recovery from the gel and optimized, then synthesized on the pET-21b (+) expression vector and registered in the NCBI database under accession number MT482301.1. The accuracy of keratinase synthesis in the expression vector was confirmed by PCR, double digestion and sequencing (Figures 2A–C). After transferring the recombinant pET-21b(+)-ker to the *E*.*coli* BL21 expression host and culturing the bacterium on ampicillin-containing LB plate agar, an expression test was performed on the grown colonies. The results of the enzyme expression test with concentrations of 0.25, 0.5, and 1 mM IPTG were evaluated and it was observed that the protein is expressed in all the above concentrations of IPTG. A band of about 38 kDa confirmed the expression of the recombinant keratinase gene on SDS-PAGE gel.

### Purification and western blot analysis of recombinant keratinase

Because the expressed protein was not removed from the inclusion body using sonication, as a result, the modified Qiagen handbook method was used to solubilize the protein. The result of this step showed that the protein was completely removed from the inclusion bodies. Then, the keratinase recombinant C-terminal His-tagged was purified effectively using the Ni-NTA column.

In the nickel affinity chromatography column, histidine-sequence proteins bind to the resin with a high affinity. After washing the other proteins from the column, with an increasing concentration of imidazole, the recombinant protein is also separated from the column. The protein was gradually separated from the column by increasing the imidazole concentration from 20 mM and above. In protein purification using a nickel affinity chromatography column and by increasing the concentration of imidazole to 20, 40, and 250 mM, the protein was gradually purified, showing the highest purity at a concentration of 250 mM imidazole. Due to the presence of urea and other salts including imidazole in purification buffers, a dialysis bag was used to replace the buffer (Figure 2D). Further, the purification steps of KRLr1 are shown in Table 1.

**Table 1:** Protein activity and concentration, purified using Ni-NTA chromatography.

Step	Volume (mL)	Total activity (U)	Total protein (mg)	Specific activity (U/mg protein)	Yield (%)	Purification (fold)
Crude extract	50 mL	620	205	3.02	100	1
Ni-NTA	4 mL	533	2.4	222.01	85.96	73.41

To confirm the presence of recombinant keratinase on an SDS-PAGE gel, western blotting was performed with primary Penta • His Antibody and secondary conjugate antibody HRP-Poly Histidin against the histidine sequence of the protein end. As shown in Figure 2E, since the secondary antibody is a binding enzyme that can act on its specific substrate and detect the protein, the reaction product appeared on the radiological film in the presence of ECL.

### Feather digestion and its amino acids compounds

**Figure 3:**
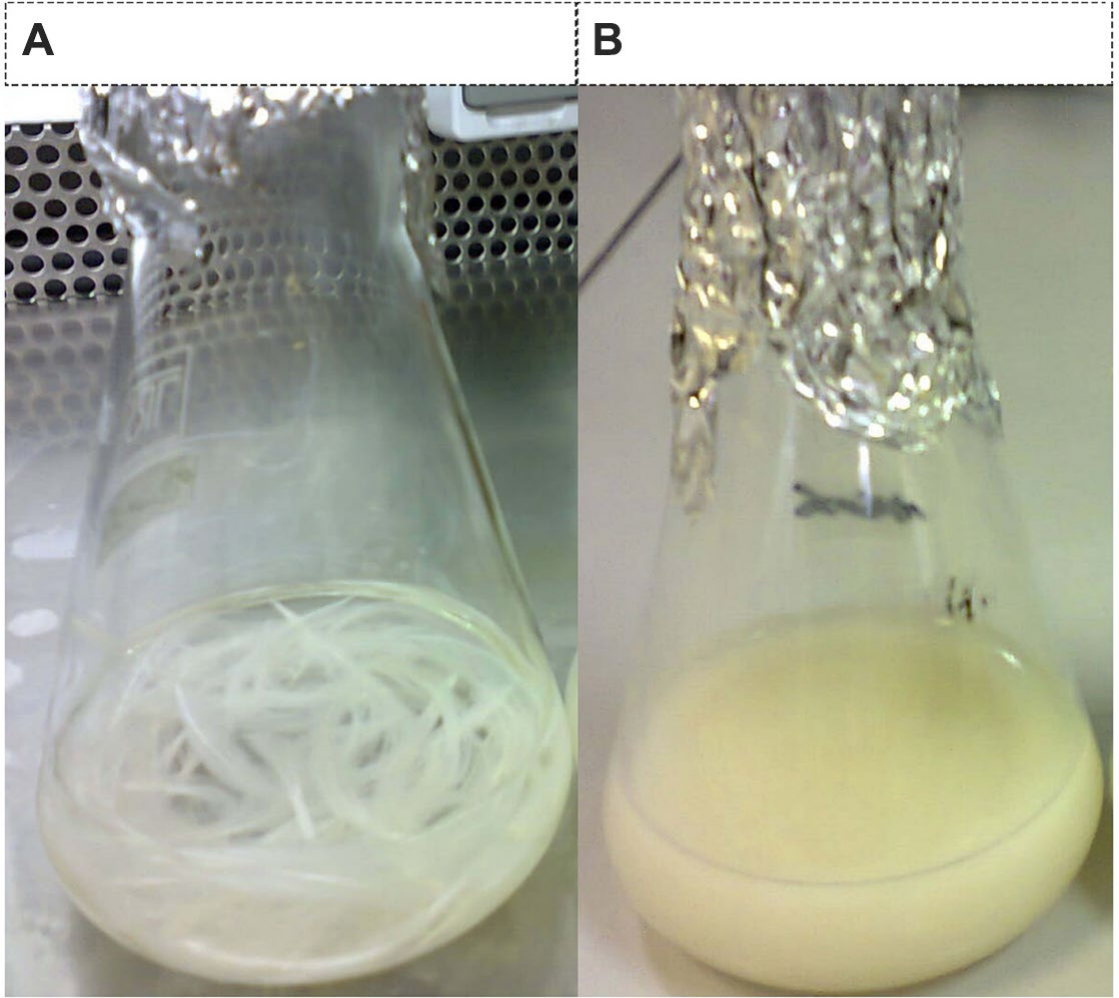
Feather digestion. **(A)** Culture medium containing enzyme-free feathers (control sample) and **(B)** feather microbial digestion using recombinant enzymes.

To investigate the digestion of the feather using the recombinant enzyme, 2 mL of the purified enzyme was added to 100 mL of FB medium containing the chopped feather (1–2 cm). After incubation to 37°C, complete digestion of the feather was observed in less than 72 h (Figures 3A,B). After the incubation period, the culture medium was first filtered using filter paper and then, using a centrifuge, the supernatant was stored as a product of digestion to further study the enzyme activity at 4°C. Analysis of amino acids from feather digestion was performed by the HPLC method. The results of this analysis are given in Table 2. As can be seen, the amino acids cysteine, phenylalanine, tyrosine and lysine contain the highest amount of amino acids.

**Table 2:** Analysis of amino acids resulting from feather digestion by the recombinant enzyme.

Amino Acids	From recombinant enzyme (g/L)
Alanine	0.019
Valine	0.016
Isoleucine	0.007
Serine	0.024
Aspartic acid	0.006
Glutamic acid	0.043
Lysine	0.088
Tyrosine	0.095
Arginine	0.000
Glycine	0.012
Leucine	0.016
Threonine	0.010
Methionine	0.012
Phenylalanine	0.115
Cystine	0.315
Histidine	0.034
Glutamine	0.000
Ornithine	0.012
Citrulline	0.026
Total	0.854

### Effect of temperature and pH on keratinase activity and stability

**Figure 4:**
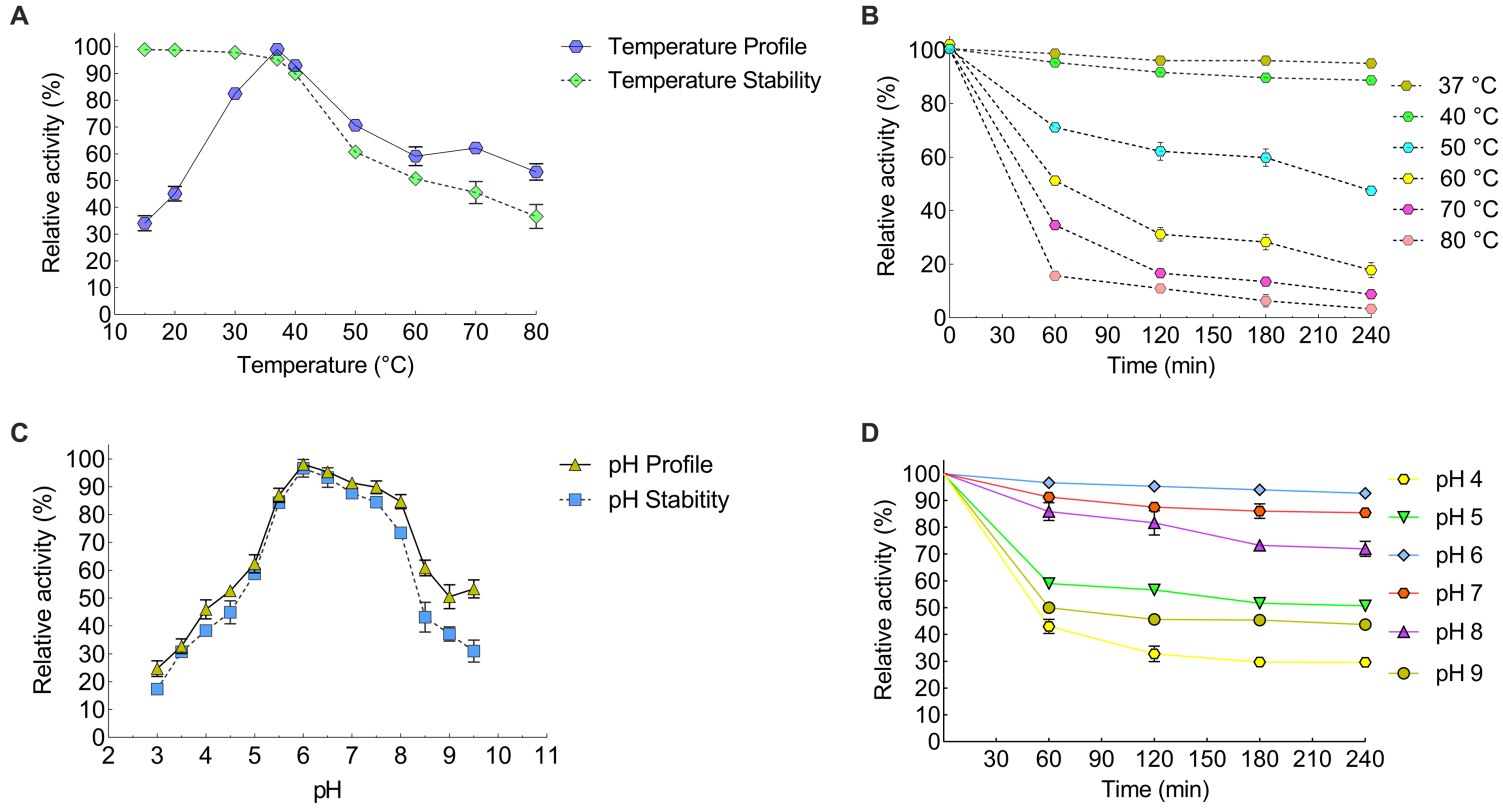
Efect of temperature and pH on the recombinant KRLr1. Efect of temperature on the recombinant enzyme **(A)** activity and survival, and **(B)** stability. Efect of pH on the recombinant enzyme **(C)** activity and survival, and **(D)** stability.

The recombinant keratinase activity was performed by the modified method of Lin and Yin (2010). This experiment was performed three times on the purified keratinase enzyme. The results of this study showed that the enzyme keratinase has an average activity of 0.885 at a wavelength of 280 nm. The diagram of relative enzyme activity at different temperatures was plotted using Prism 6 software (Figures 4A,B; Table 3). The results showed that the enzyme is most active at 37°C and its optimal activity is at 37°C. The effect of pH 3–10 on the activity of the keratinase enzyme was measured three times at 37°C. A diagram of the relative activity of the enzyme was drawn at different pHs (Figures 4C,D). The results showed that the enzyme is active at pH 6–8 (more than 60% of the activity) and the optimal pH for the activity of the enzyme is pH 6.

**Table 3:** Functional comparison of KRLr1 with other enzymes of the same family.

Family	Host	Substrate	Opt. Temperature	Opt. pH	Purification (fold)	Activity	K_m_	K_cat_	k_cat_/K_m_	References
Serine peptidase/subtilisin-like S8 family	*Bacillus licheniformis*	Keratin (degraded feather)	37°C	6	73.41	533 U/mL	14.54 mM	912.7 S^−1^	62.77 M^−1^ S^−1^	This study
Serine protease	*Bacillus licheniformis*	Degraded feather, bovine serum albumin, casein, gelatin	40°C	10.5	–	323 U/mL	–	–	–	(Liu et al., 2013)
Protease	*Bacillus licheniformis* S90	Keratin azure	50°C	7.5		195 U/mL to 324 U/mL	–	–	–	(Hu et al., 2013)
Protease	*Bacillus licheniformis*		50°C	7.5	70		–	–	–	(Lin et al., 1992)
Protease	*Bacillus licheniformis* MKU3	Keratin azure, azocasein	–	–	–	166·2U/mL^−1^	–	–	–	(Radha and Gunasekaran, 2007)
Keratinase	*Bacillus licheniformis* PWD-1	Azocasein, bovine serum albumin, collagen, and soy protein	55–60°C	8.5–9.5	–	285 U/mL^−1^	–	–	–	(Porres et al., 2002)
Keratinase	*Bacillus* sp. RCM-SSR-102	Feather	50°C	10	–	–	–	–	–	(Kshetri et al., 2020)
Thermo-and-solvent (DMSO) stable serine keratinase	*Bacillus pumilus* AR57	Feather	45°C	9	–	37 U/mL	–	–	–	(Jagadeesan et al., 2020)
Serine protease	*Bacillus* sp. NKSP-7	Keratin	65°C	7.5	3.02-fold	51.50 U/ mL^−1^	1.13 mg/mL^−1^	2316.4 s^−1^	2049.91 mL/mg^−1^ s^−1^	(Akram et al., 2020)
Keratinase	*Bacillus* sp. CSK2	Chicken feathers	60–80°C	8	–	539.09 U/mL	–	–	–	(Nnolim and Nwodo, 2020)
Keratinase	*Bacillus tropicus* Gxun-17	Keratin	60°C	7	3.18-fold	112.57 U/mL	15.24 mg/mL	–	–	(Shen et al., 2022)
Keratinase	*Bacillus pacificus* RSA27	Feather	60°C	9	2.58-fold	38.73 U/mg	5.69 mg/mL	–	–	(Sharma et al., 2022)

### Effects of metallc ions, inhibitors, organic solvent, and surfactants on the activity of KRLr1

**Figure 5:**
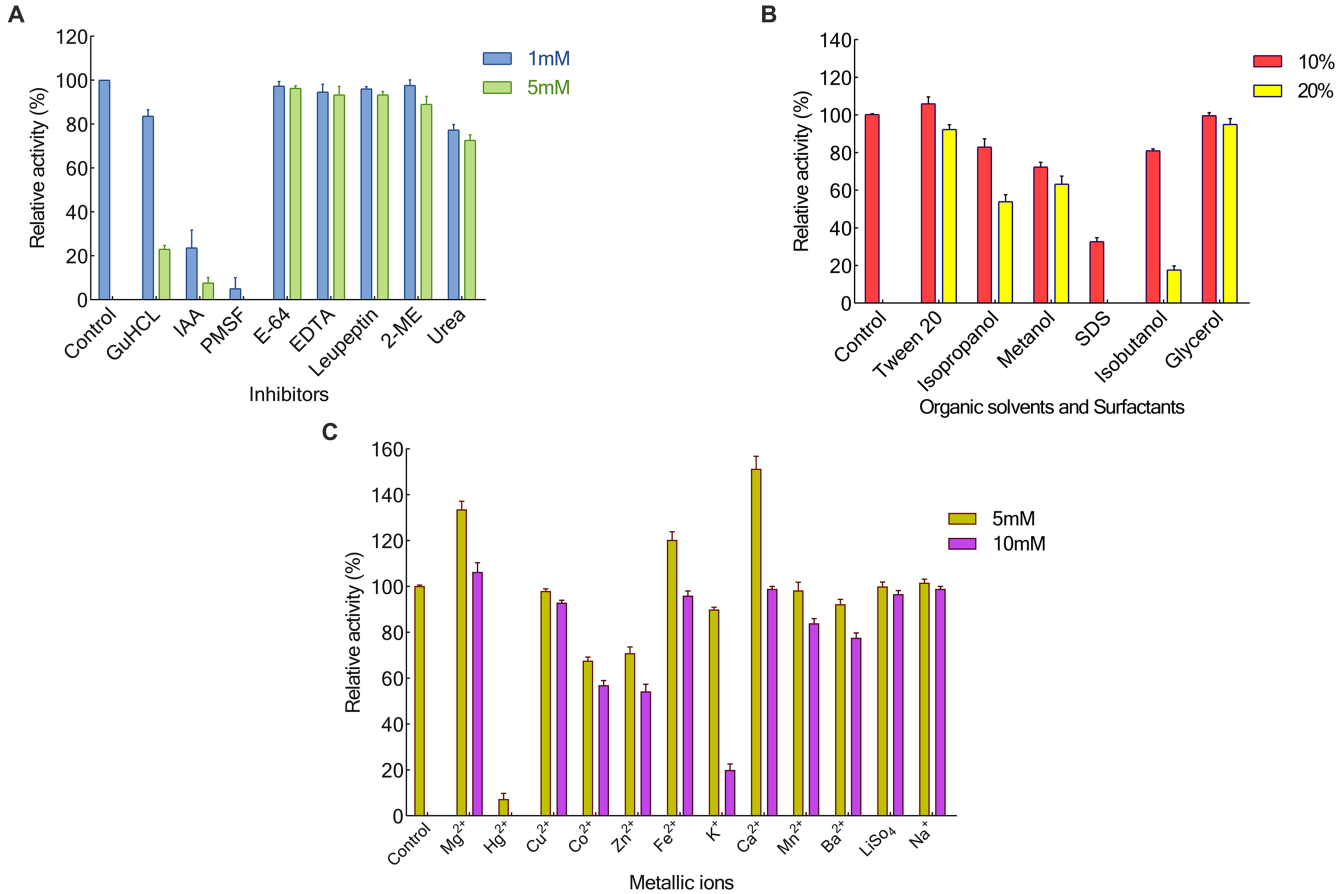
Effects of Inhibitors **(A)**, Organic solvants and Surfactants **(B)**, also Metallic ions **(C)** on KRLr1 activity.

We looked into a variety of potential metallic ions or inhibitors of keratinase to evaluate their impact on KRLr1’s activity (Figures 5A–C). In this experiment, the majority of tested metal ions had no a notable restraining effect, except for Hg^+^ and K^+^(5 and 10 mM). Nevertheless, the addition of Fe^2+^ and Ca^2+^ at 5 mM, and Mg^2+^ at both of its tested concentrations, caused a slight increase in the activity of KRLr1. PMSF, the most effective of the specific enzyme inhibitors tested, showed the strongest inhibition of the enzyme. Whereas increasing the concentrations of other keratinase inhibitors (such as E-64 and Leupeptine) up to 5 mM had no a significant inhibitory effect. Also, GuHCl had the most strong inhibitory effect on the KRLr1 among the general enzyme inhibitors that were tested, while 2-ME and urea did not affect enzyme activity. SDS was the sole surfactant examined that totally impeded the enzyme’s functioning. The KRLr1 activity was augmented in the presence of 10% surfactants of Tween20. The KRLr1 was observed to be able to tolerant the remarkable amount of organic solvents, and its performance was improved up to 10% with the addition of glycerol.

### Thermodynamic analysis of the KRLr1

**Figure 6:**
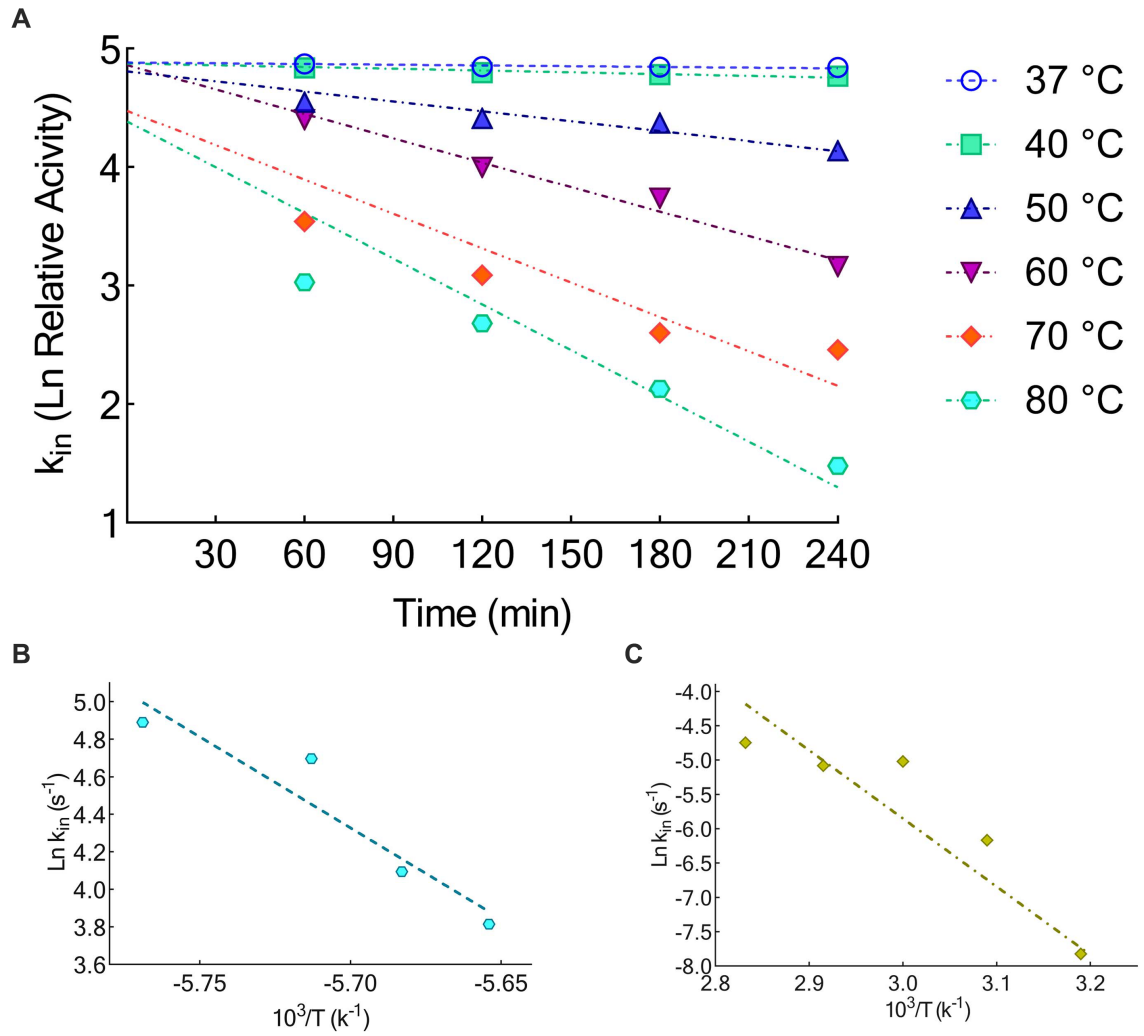
**(A)** Thermal inactivation Ln at 37, 40, 50, 60, 70, and 80°C. **(B)** Arrhenius plots for Ea^‡^. **(C)** Arrhenius plots for Ea^#^. The graphs were plotted by using GraphPad Prism V.8.

The activation energy of KRLr1 was calculated using the Arrhenius equation (Figure 6A). Table 4 displays the thermodynamic parameter values. The fact that the KRLr1 requires 28.15 kJ/mole energy to activate the reaction (E_a_^‡^) shows that KRLr1 have a quick reaction. KRLr1 reacts more quickly when its ΔG^‡^ levels are low.

**Table 4:** Thermodynamic parameters for the activation energy of KRLr1.

Parameters	E_a_^‡^ (kJ mol^−1^)	ΔG^‡^ (kJ mol^−1^)	ΔH^‡^ (kJ mol^−1^)	ΔS^‡^ (kJ mol^−1^)	ΔG^‡^_(E-T)_ (kJ mol^−1^)	ΔG^‡^_(E-S)_ (kJ mol^−1^)	K_a_ (1/K_m_)
Value	28.15	54.76	30.83	77.54	−10.66	6.91	0.068

The parameters were measured after 240 min incubation at various temperatures, and the values were obtained at 37°C.

The significant values of ΔH^‡^ and ΔS^‡^ at the optimal temperature reveal the effective transition state of KRLr1. Once we see Table 4, it is realized the modest amount of energy can create the KRLr1 TS at 37°C, which supports the idea of the reaction being spontaneous, as shown by lower values of ΔG^‡^_E − T_ (− 10.66) compared to ΔG^‡^_E − S_ (6.91).

At the optimum temperature, the KRLr1 showed a very low k_in_ while having a high t_1/2_ (Figures 6B,C). The highest t_1/2_ was obtained at 37°C, but a considerable decrease was observed in the t_1/2_ of higher temperatures.

Due to KRLr1’s high E_a_^#^ value (82.58 kJ/mol), irreversible thermal inactivation of this keratinase requires a significant amount of energy. The amount of ΔG^#^ for KRLr1 rose as the temperature increased, indicating both the transition state that happens later and the enzyme’s resistance to irreversible heat inactivation and denaturation. For thermal deactivation, entropy (ΔS^#^) and enthalpy (ΔH^#^) changes showed decreasing patterns as temperature increased from 37 to 80°C.

The optimal temperature’s lower ΔH^#^ and ΔS^#^ values indicated that KRLr1 is more thermally stable in activity optimal temperature than it is at higher temperatures. KRLr1 values for ΔS^#^, ΔH^#^, and ΔG^#^ showed that this keratinase enzyme has a stable conformation at the optimum activity temperature. The thermodynamic characteristics of KRLr1 are listed in Table 5.

**Table 5:** Thermodynamic parameters for irreversible thermal inactivation of KRLr1.

Temp	k_in_ (m^−1^)	t_1/2_ (min)	E_a_^#^ (kJ/mol)	ΔH^#^ (kJ/mol)	ΔG^#^ (kJ/mol)	ΔS^#^ (JmolK^−1^)
37°C	0.02 × 10^−2^	1732	82.58	80.00	46.65	107.00
40°C	0.04 × 10^−2^	866		79.97	46.73	106.43
50°C	0.21 × 10^−2^	165		79.89	47.30	101.21
60°C	0.66 × 10^−2^	55		79.81	48.12	95.72
70°C	0.62× 10^−2^	52		79.72	49.28	89.53
80°C	0.87× 10^−2^	39		79.64	50.91	84.79

The parameters were measured after 240 min incubation at various temperatures.

### Bioinformatics studies

#### *In silico*, theoretical-biochemical and phylogenetic analysis of keratinase KRLr1

**Figure 7:**
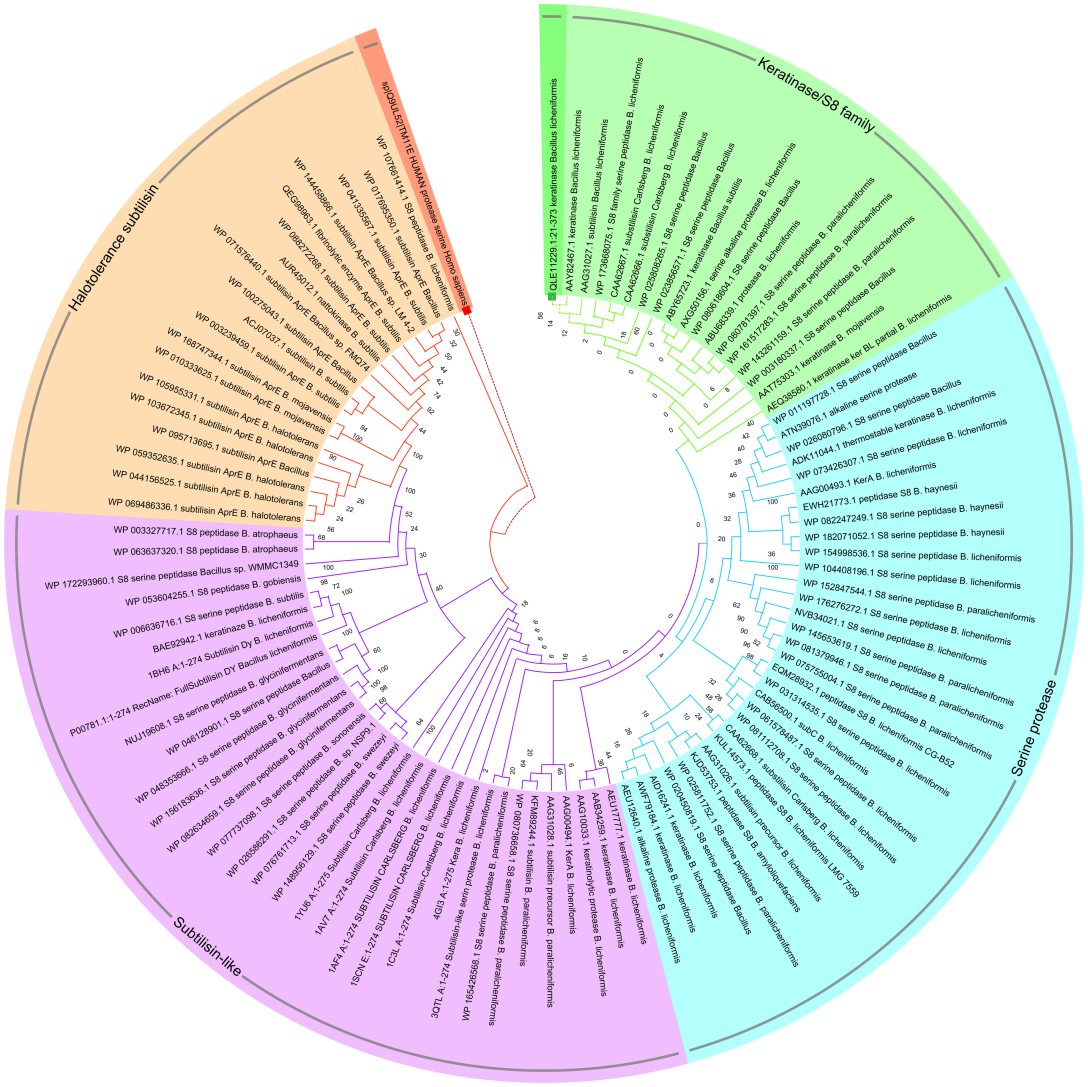
Circular phylogenetic relationship of keratinase KRLr1 from *Bacillus licheniformis* and its similar sequences. The unrooted neighbor-joining (NJ) tree was constructed based on the alignment of the protein sequences with the high similarities. The keratinase KRLr1 is marked with a green box. The amino acid sequence of human serine protease was used as an out-group and marked with a red box.

The translation of nucleotide sequences to amino acid sequences in the Expasy translation tool resulted in a protein with 353 aa and a molecular weight of 35.96. Computation of the Instability index (II) of equal 12.20, Aliphatic index (AI) of 5.69, and Grand average of hydropathicity (GRAVY) of −0.021 are confirming that this keratinase is classified as a stable protein. Circular phylogenetic tree analysis of amino acid sequences KRLr1 showed the highest similarity to *Bacillus licheniformis* keratinase that belongs to serine peptidase/subtilisin-like S8 family (Figure 7).

**Figure 8:**
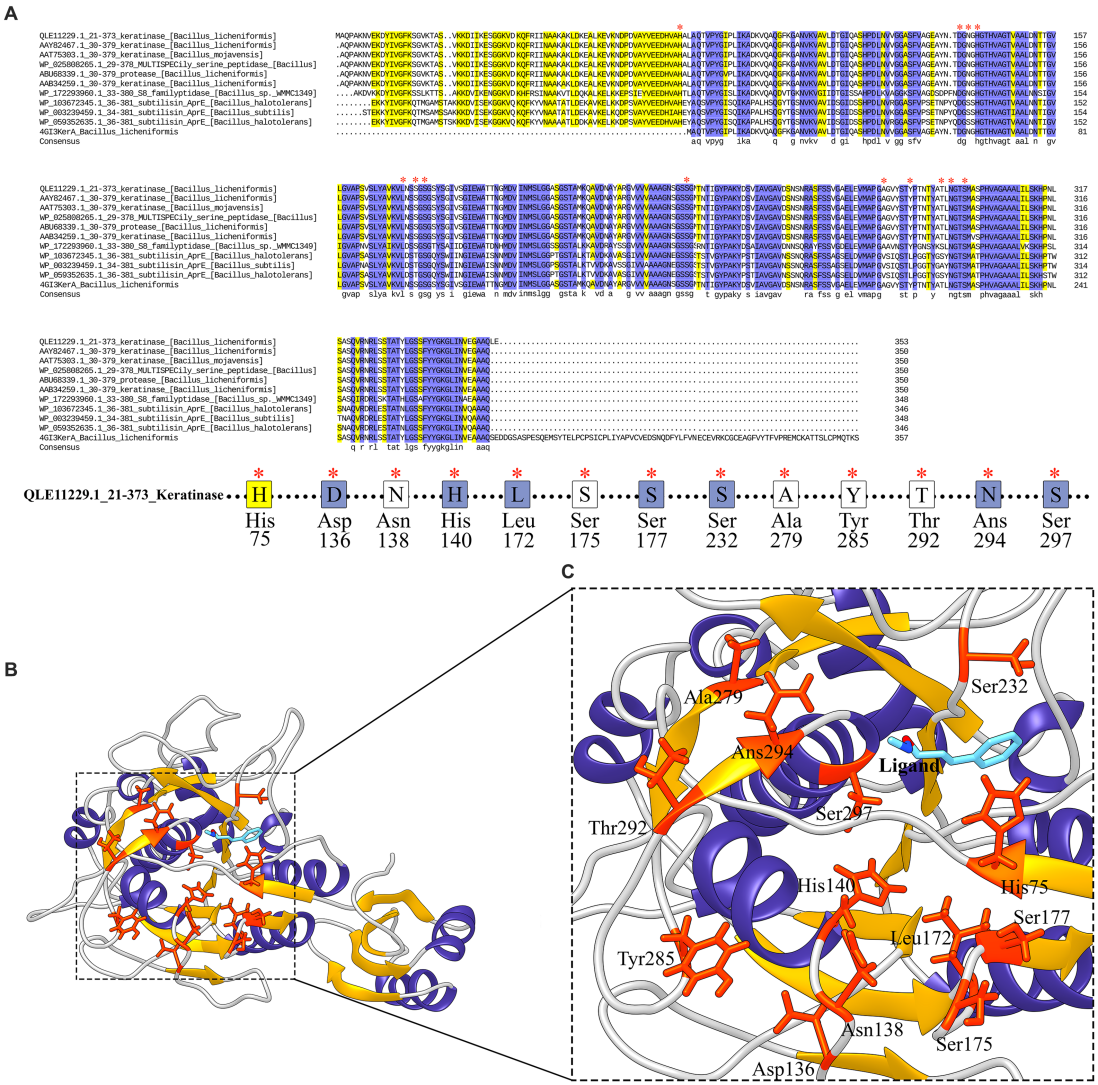
Conserved amino acid sequence and predicted active site of keratinase KRLr1. **(A)** Protein sequence alignment of the keratinase KRLr1 and several closely related sequences. The * over residues represent the conserved active site amino acids. **(B)** Modeled 3D structure of keratinase KRLr1. **(C)** The amino acids involved in the catalytic site of keratinase KRLr1 (His75, Asp136, Asn138, His140, Leu172, Ser175, Ser177, Ser232, Ala279, Thr292, Ans294, and Ser297) are shown in orange-red ribbon sticks and the ligand is represented in a blue stick. The α-helixes are shown as purple ribbon, β-sheets are shown as golden ribbon, and coils are shown as the gray rod.

The S8 family is a large class of serine proteases that are found in Archaea, Bacteria, fungi, yeasts, and eukaryotes. Their catalytic activity is similar to the trypsin family of serine proteases which gets benefits from catalytic triad including aspartic acid, serine, and histidine residues involved in the catalytic domain, However, the active site residues frequently occur in the Asp-Thr/Ser-Gly, His-Gly-Thr-His, and Gly-Thr-Ser-Met-Ala-Xaa-Pro (subfamily S8) or Asp-Asp-Gly, His-Gly-Thr-Arg, and Gly-Thr-Ser-Ala/Val-Ala/Ser-Pro motifs (subfamily S8B). This property could be considered as signatures specific to that category of the S8 family. Commonly S8 protein structure is formed through three layers with a seven-stranded β sheet sandwiched between two layers of helices. According to these results, keratinase KRLr1 belongs to serine peptidase family S8. As shown in Figure 8A, most catalytic triad amino acids are locating in conserved regions keratinase. Furthermore, active site amino acids were predicted using the COFACTOR server (Figure 8B). A specific ligand is magnified in the KRLr1 catalytic cleft (Figure 8C).

Keratinase initial signal peptide was removed during the design construct and SignalP suggesting the intracellular production of the keratinase.

#### Homology modeling, refinement, and validation of KRLr1 structure

**Figure 9:**
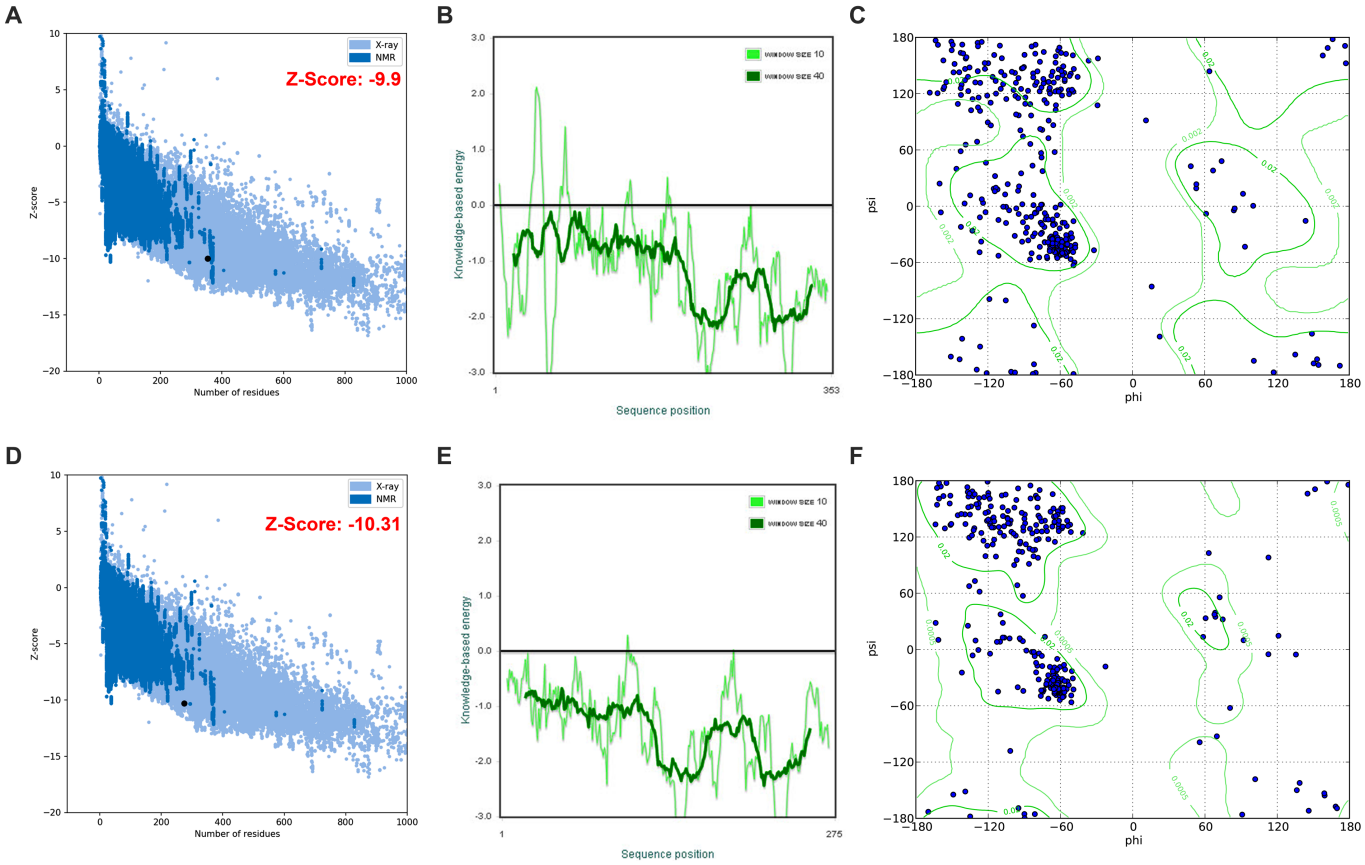
The validation of homology modeling results. **(A)** Before and **(B)** after of ProSA Z-score analysis for homology modeled keratinase KRLr1. The plot showed that the black spot (predicted model) with the Z-Score value of −9.9 is within the range of native conformation of all protein chains in PDB determined by X-ray crystallography spectroscopy concerning their length. **(C)** Before and **(D)** after of ProSA average energy of modeled protein. The bold green curve is related to the negative energy of the bunches of 40 amino acids. The pale green curve corresponds to the negative energy of the bunches of 10 amino acids. **(E)** Before and **(F)** after Ramachandran’s graph analysis for homology modeled keratinase KRLr1. Ramachandran’s graph analysis depicts that 98% of residues fall within the favored and allowed regions, while the outlier region represents only 2%.

The highest keratinase KRLr1 structure similarity was shown with Chain A, subtilisin *Bacillus subtilis* (RCSB code: 3WHI) through PDB-Blast NCBI (Maynes et al., 2005). Keratinase KRLr1 PDB was obtained from MODELLER V.9 with assuming of 3WHI PDB as a template structure. KRLr1 PDB structure was refined by the ModRefine server and validation of the modeled KRLr1 was surveyed in PROSA and PROCHECK servers. The Z-score output was estimated to equal −10.31 after KRLr1 PDB refinement (Z-score was equal −9.9 before refinement), this indicates that the KRLr1 structure was well modeled in X-ray areas (Figures 9A,B). Furthermore, the negative energy of modeled KRLr1 is improved after refinement (Figures 9C,D). The Verify-3D scores and ERRAT quality factors were estimated at 58 and 93.06%, respectively. Based on Ramachandran graph results, 90% of the amino acids were in the favored, 8% in the allowed, and 2% in the outlier regions after refinement which indicates that the amino acids KRLr1 are well-positioned at the angles phi (φ) and psi (ψ) (Figures 9E,F). The validation results show that KRLr1 has been properly modeled.

**Figure 10:**
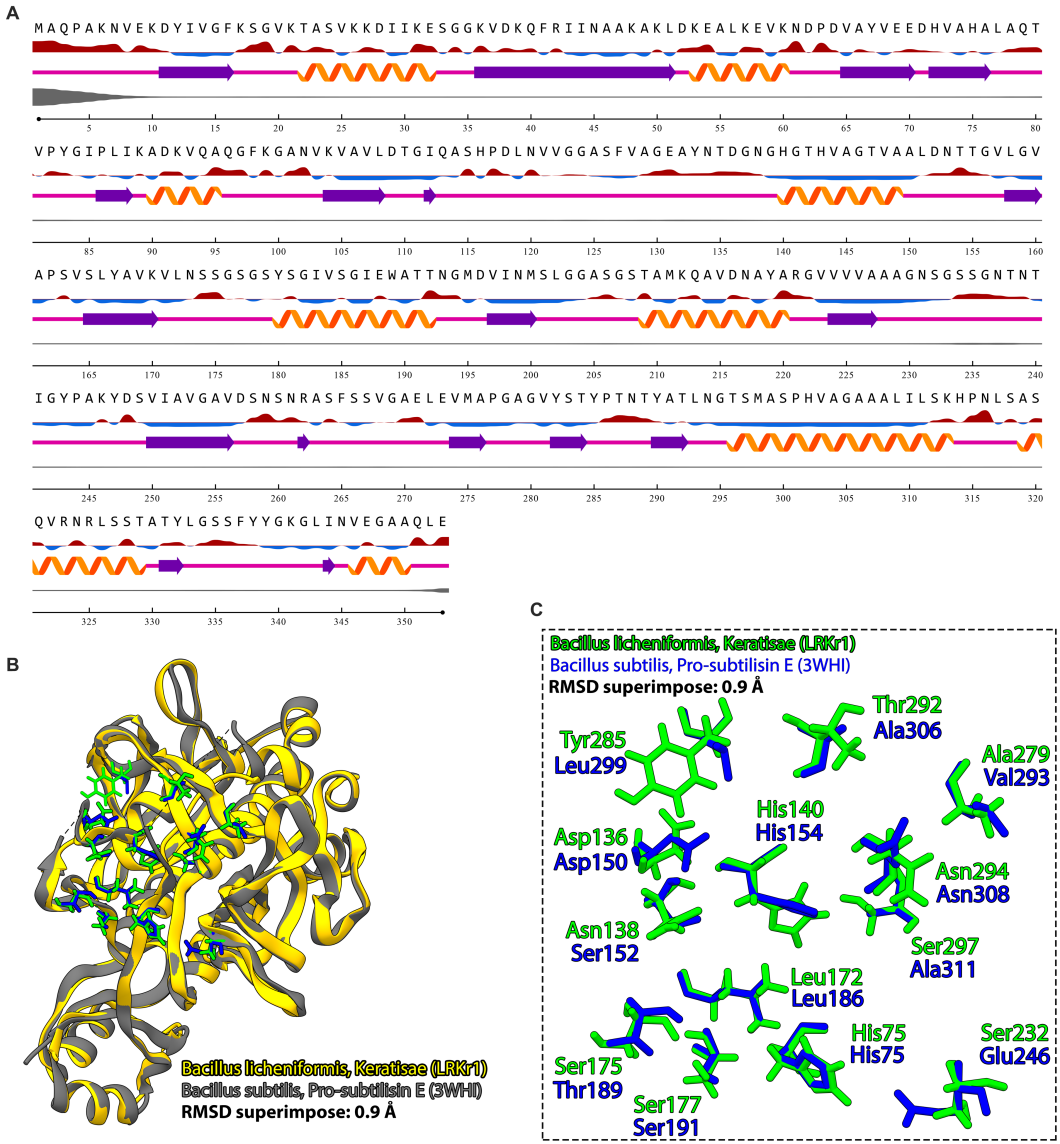
Prediction of secondary structure and PDB superimposition. **(A)** Predicted keratinase KRLr1 secondary structure by NetSurfP-2.0. The α-helixes are purple and β-sheets are orange. **(B)** The PDB superimposition of keratinase KRLr1 (yellow) with PDB of *B. subtilis* subtilisin (3WHI) (gray) at RMSD of 0.9A. **(C)** Magnified superimposition active site cleft of keratinase KRLr1 with *B. subtilis* subtilisin (3WHI). Active site residues of keratinase KRLr1 are shown as the green sticks and active site residues of *B. subtilis* subtilisin (3WHI) are shown as blue sticks.

The results of the NetSurfP-2.0 server showed that the KRLr1 consists of a secondary structure containing 25% α-helix and a 27% β-sheet (Figure 10A). The amino acids predicted for the active site cleft of KRLr1 are located close to the structure of *Bacillus subtilis* subtilisin E (Figure 10B). These amino acids as follows: His75, Asp136, Asn138, His140, Leu172, Ser175, Ser177, Ser232, Ala279, Thr292, Ans294, and Ser297 for KRLr1 which are shown in orange sticks. Using the superimposition command in Chimera V13.1, the modeled structure of the KRLr1 was compared with three high-similar structure relative to the *Bacillus subtilis* subtilisin E (Figure 10C).

#### Protein–protein docking and the HADDOCK results analysis

Because KRLr1 was able to digest chicken feather keratine (as substrate) *in vitro*, the gene encoding chicken feather keratine 4 and feather keratine 12, located on chromosome 2 (Chr2_FK4) and chromosome 25 (Chr25_FK12), respectively, were retrieved and theirs 3D structures were predicted and modeled by the I-Tasser online server. Cleavage sites on the amino acid sequence of chicken FK4 and FK12 were estimated using the PeptideCutter server in Expasy. Due to the functional similarity of KRLr1 with serine proteases, sites cleavaged on the FK4 and FK12 amino acid sequences by trypsin were considered for in this step.

**Figure 11:**
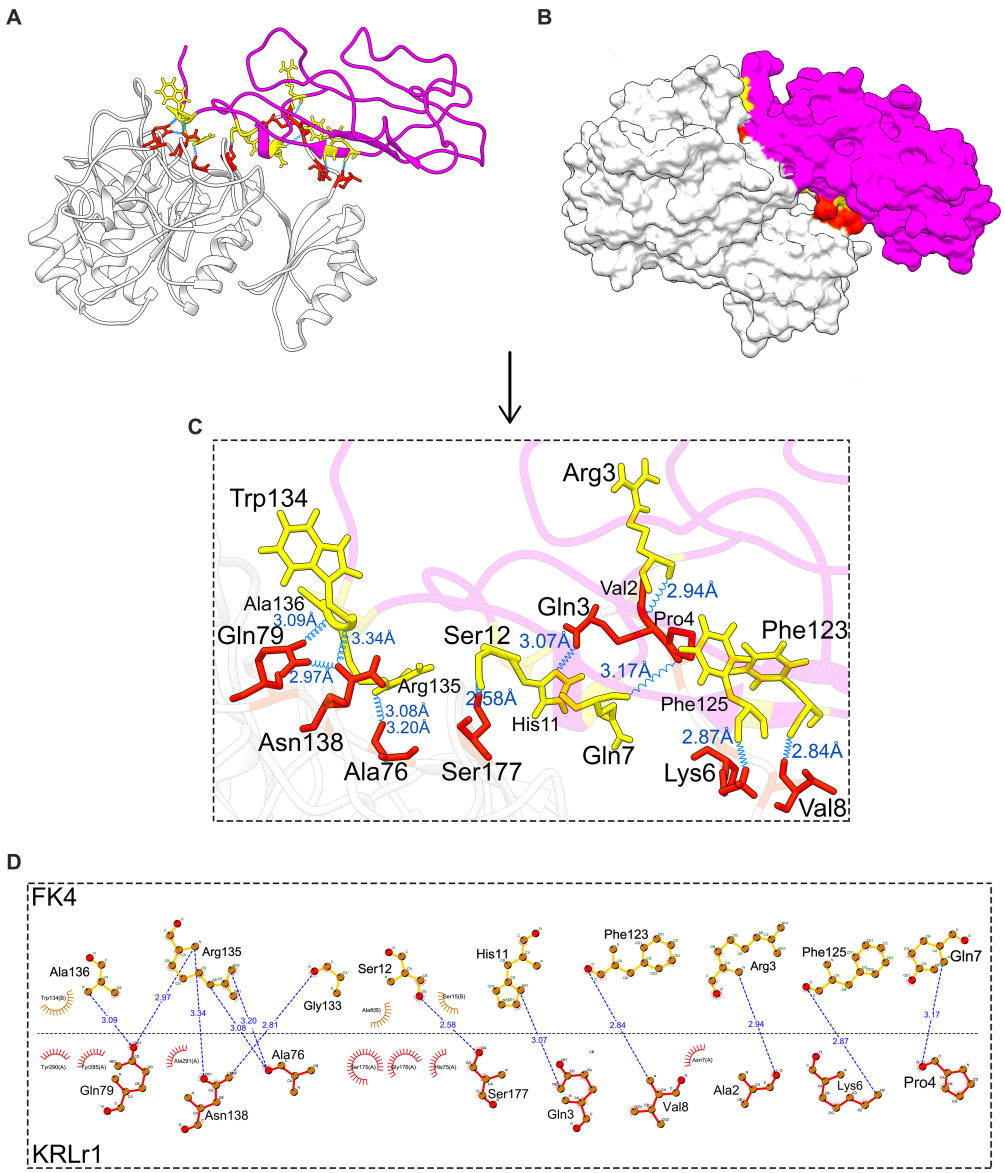
Result of protein–protein docking of keratinase KRLr1 and feather keratine 4 (FK4) complex after 50 ns of MD simulation. **(A)** Docked KRLr1 and FK4 complex in ribbon and **(B)** surface view. The KRLr1 is gray-white and FK4 is purple. **(C)** The magnification of KRLr1 and FK4 complex amino acids which are involved in the complex by hydrogen. Amino acids related to KRLr1 and FK4 are shown as orange-red and yellow sticks, respectively, bond with a 45° turn right-rotation view. Hydrogen bonds are represented as a blue wave. **(D)** The 2-D display of interacted amino acids within KRLr1 and FK4 complex by LigPlot+. The hydrogen bond is represented as a blue dashed line.

**Figure 12:**
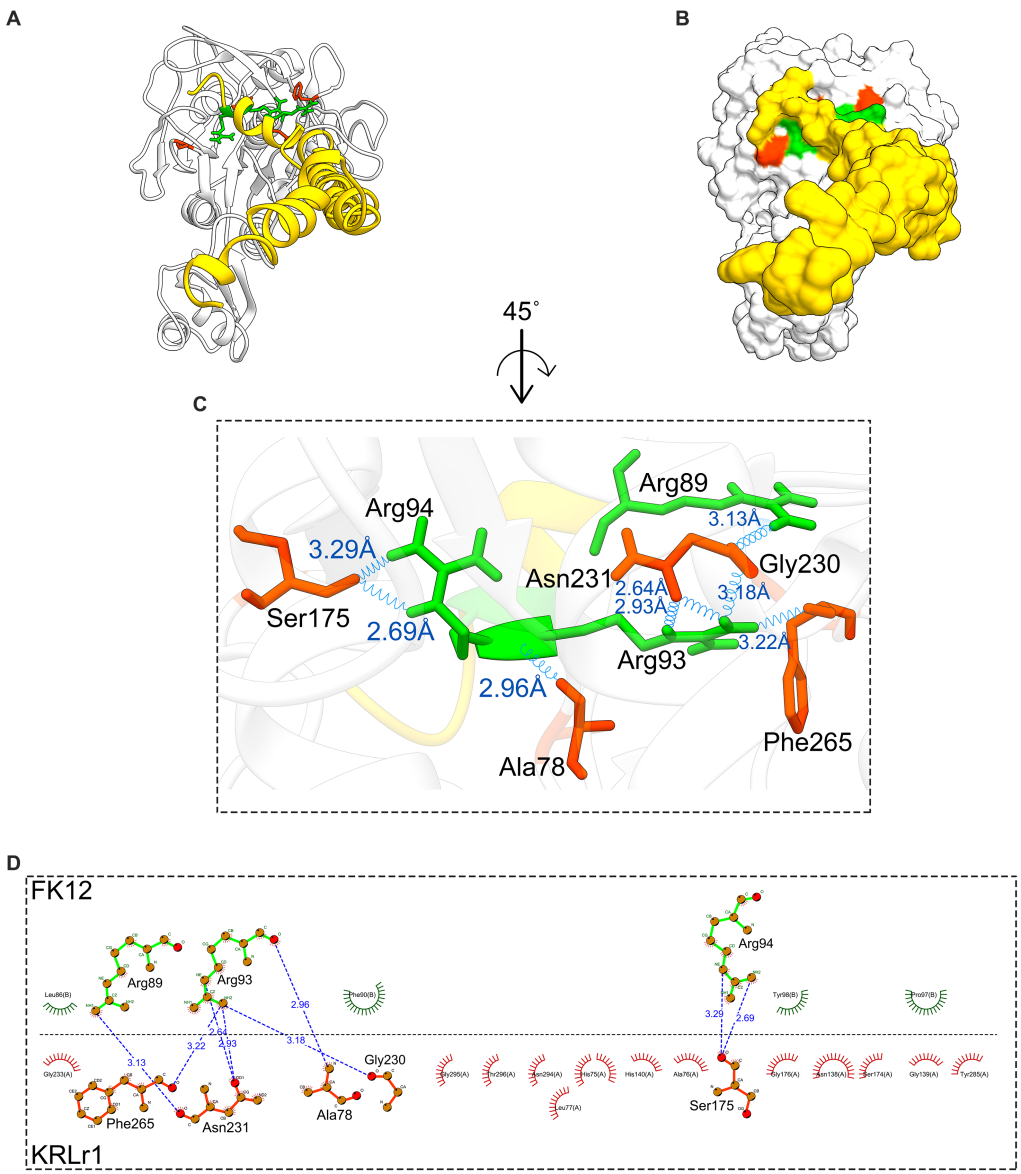
Result of protein–protein docking of keratinase KRLr1 and feather keratine 12 (FK12) complex after 50 ns of MD simulation. **(A)** Docked KRLr1 and FK12 complex in ribbon and **(B)** surface view. The KRLr1 is gray-white and FK12 is golden. **(C)** The magnification of KRLr1 and FK12 complex amino acids which are involved in the complex by hydrogen. Amino acids related to KRLr1 and FK4 are shown as orange-red and green sticks, respectively, bond with a 45° turn right-rotation view. Hydrogen bonds are represented as a blue wave. **(D)** The 2-D display of interacted amino acids within KRLr1 and FK12 complex by LigPlot+. The hydrogen bond is represented as a blue dashed line.

After the preparation of KRLr1, FK4, and FK12 in the Chimera V13.1, the protein structures of all three were stored as PDB. The KRLr1-FK4 and KRLr1-FK12 structures were then introduced to the HADDOCK server for docking. The best complexes were selected from the cluster with the lowest binding energy equal to −140 and −111 kcal/mol (Figures 11A–D, 12A–D; Supplementary Figure S1).

The HADDOCK statistical results of KRLr1-FK4 and KRLr1-FK12 complexes were compared. The HADDOCK Scores with the highest negative binding energy from clusters relative to the KRLr1-FK4 and KRLr1-FK12 complexes were selected. Overall HADDOCK results showed that LRRk1 could interact with FK4 (−130.7 ± 1.5 kcal/mol) with higher negative energy than FK12 (−111.1 ± 22 kcal/mol).

#### MD results

The changes in amino acid interactions of KRLr1-FK4 and KRLr1-FK12 complexes after 50 ns of simulation by MD. In both complexes were formed Ser-Glu or Ser-Gly dyad. An increase in the hydrogen bond number for KRLr1-FK4 has been observed compared to the KRLr1-FK12 complex. However, the maintenance of the hydrogen bond between the important amino acids serine12 and serine177 after 50 ns MD simulation indicates that the active site of KRLr1 has interacted with the FK4 protein. These results also show that the serine 175 in the KRLr1-FK12 complex, as a key amino acid of the active site of KRLr1, still maintains its hydrogen bond after 50 ns.

**Figure 13:**
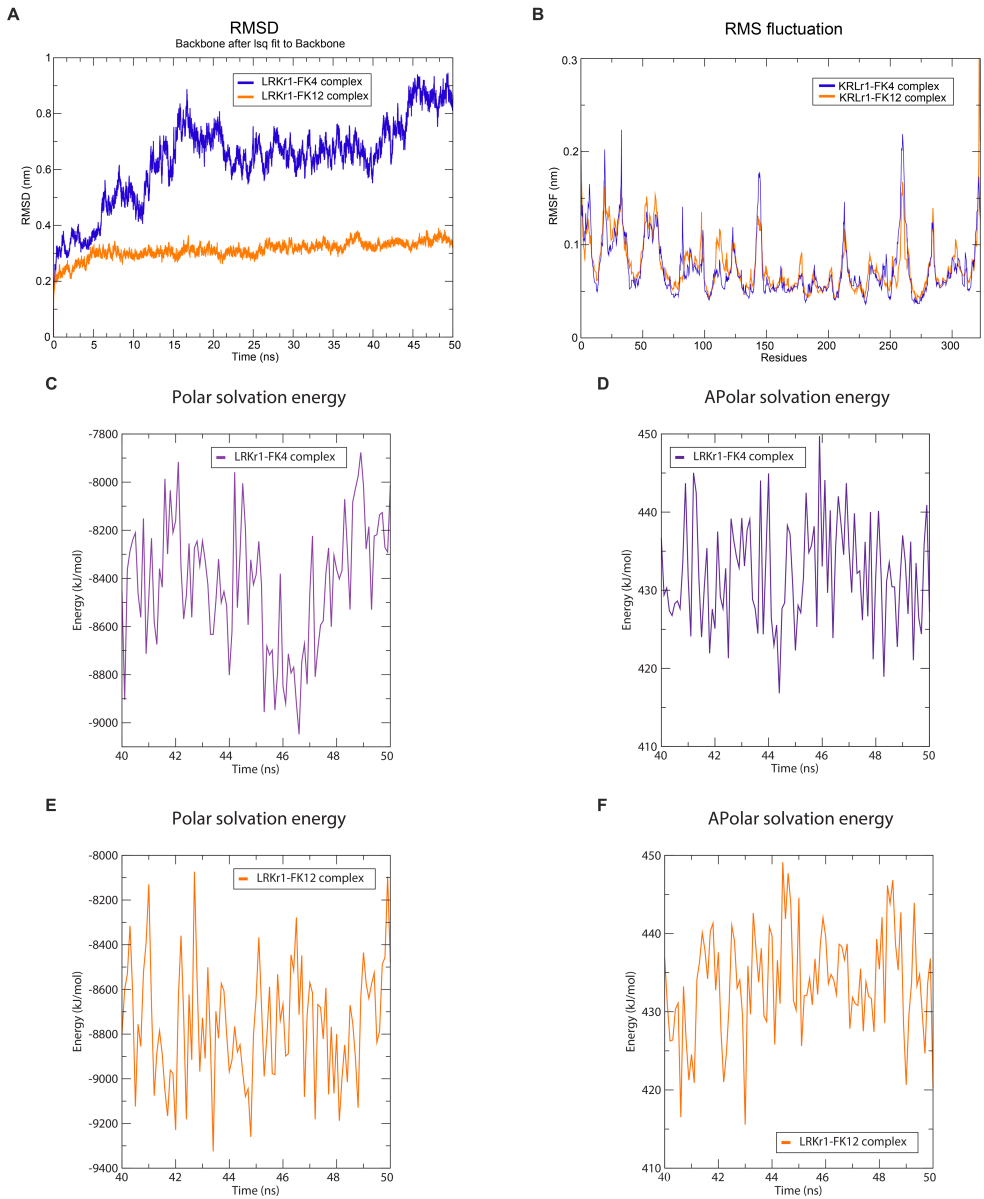
Molecular dynamics simulation results. **(A)** RMSD graph of the KRLr1-FK4 complex (purple) and KRLr1-FK12 complex (orange). **(B)** RMSF graph of the KRLr1-FK4 complex (purple) and KRLr1-FK12 complex (orange). **(C)** Polar solvation energy and **(D)** Apolar solvation energy of KRLr1-FK4 complex. **(E)** Polar solvation energy and **(F)** Apolar solvation energy of KRLr1-FK12 complex.

**Figure 14:**
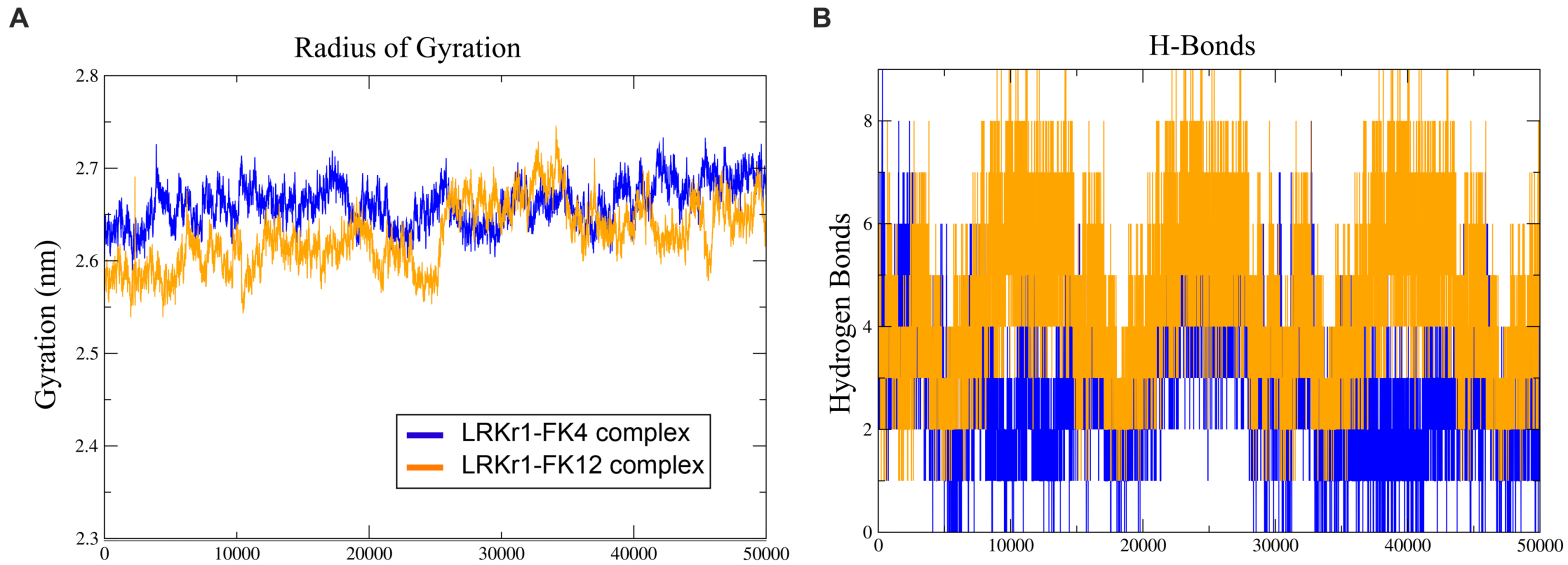
**(A)** Radius of gyration. **(B)** H-Bonds formation for Interactions.

The overall RMSD of the docked of KRLr1-FK4 complex (0.7 nm) was in the higher value compared to the and KRLr1-FK12 complex (0.3 nm) (Figure 13A). This finding suggests that more of the amino acids in the active site of KRLr1 are involved with the FK4 protein. Root Mean Square Fluctuation (RMSF) plot indicated a simulation structural fluctuation profile in the active site of KRLr1 in complex with FK4 compared to the KRLr1-FK12 complexes (Figure 13B). Besides, it is observed that the increase in RMSF is related to the KRLr1-FK12 complex in confirming the RMSD results because it indicates an increase in the interaction between KRLr1 and FK4. However, the gyration and H-Bond diagrams of each of the complexes show their reasonable stability (Figures 14A,B).

#### MM/PBSA analysis

Calculation of binding free energy of the MD results was performed by MM/PBSA analysis. Polar and apolar parameters related to the KRLr1-FK4 and KRLr1-FK12 complexes was determined through MM/PBSA calculation (Figures 13C–F). Furthermore, based on the 50 ns MD of the KRLr1-FK4 and KRLr1-FK12 complexes, binding free energy analysis and its corresponding parameters were analyzed by the MM/PBSA calculation and reported in Table 5. The results showed that the complex of KRLr1-FK4 exhibited a relatively binding free energy value of −266.4 ± 61.28 kJ/mol as compared to the KRLr1-FK12 with a free energy value of −265.36 ± 37.38 kJ/mol. Nevertheless, the Van der Wals energy of the KRLr1-FK4 complex was more negative than the KRLr1-FK12 complex, which indicates is following the MD results. Furthermore, the polar parameter in the KRLr1-FK4 complex is less positive than the KRLr1-FK12 complex that promotes negative energy of the KRLr1-FK4 complex (Table 6).

**Table 6:** Binding free energies and their corresponding components calculated from MMlPBSA analysis of the KRLr1-FK4 and KRLr1-FK12 complexes.

Energy contribution	KRLr1-FK4 complex (kJ/mol)	KRLr1-FK12 complex (kJ/mol)
ΔE_vdW_	−458.91 ± 34.00	−328.85 ± 19.79
ΔE_elec_	−290.58 ± 64.68	−513.44 ± 50.52
ΔG_polar_	537.75 ± 76.34	618.49 ± 44.16
ΔE_SAS_	−54.66 ± 6.13	−41.56 ± 2.277
ΔG_binding_	−266.4 ± 61.28	−265.36 ± 37.38

ΔEvdW, van der Waals interaction energy; ΔEelec, electrostatic interaction energy; ΔGpolar, polar solvation energy; ΔE_SAS,_ solvent-accessible surface energy.

**Figure 15:**
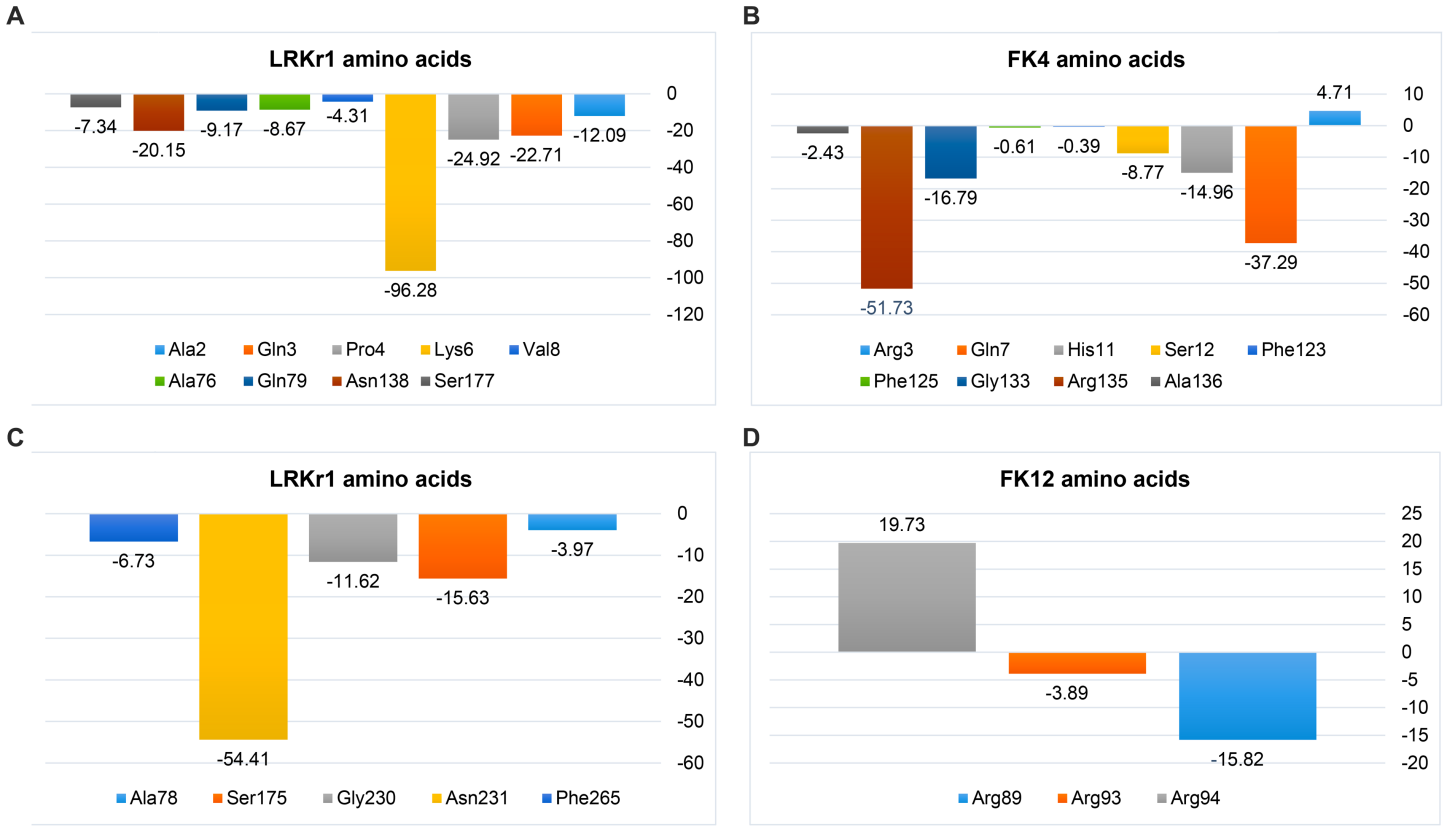
MM/PBSA output of binding free energy contribution. **(A)** The binding energy contribution of KRLr1 amino acids. **(B)** The binding energy contribution of FK4 amino acids in the KRLr1-FK4 complex. **(C)** The binding energy contribution of KRLr1 amino acids. **(D)** The binding energy contribution of FK12 amino acids in the KRLr1-FK12 complex.

The negative energy contribution of residues involved in the KRLr1-FK4 and KRLr1-FK12 complexes was determined and compared in Figure 12. The negative energy of KRLr1 and FK4 residues involved in the KRLr1-FK4 complex was determined after 50 ns MD simulation, separately (Figures 15A,B), and the negative energy of KRLr1 and FK12 residues involved in the KRLr1-FK12 complex was determined after 50 ns MD simulation, separately (Figures 15C,D). High negative energy increasing can be seen in the KRLr1-FK14 complex at the end of 50 ns MD simulation compare to the KRLr1-FK12 complex MD simulation.

These results suggest that the affinity for KRLr1 binding to FK4 is probably related to the protein structure of FK4 because more amino acids from the FK4 could be made available to the enzyme in comparison to FK12 protein.

## Discussion

Recently, microbial proteases have been of great interest to researchers due to their high activity range and application in various industries. They are the largest group of enzymes with protein hydrolytic activities such as proteinase, amidase and peptidase activity, which account for more than 60% of commercial enzymes (Liya et al., 2023). Hydrolysis is a reaction that, along with water, breaks down polymer molecules into monomers. Chicken feather keratin is a long biopolymer molecule with disulfide bonds, which is the third most abundant polymer in nature after cellulose and chitin (Onifade et al., 1998). More than 90% of the nutritional composition is composed of keratin with a molecular weight of approximately 10,500 daltons (Alahyaribeik and Ullah, 2020). Initially, two methods were used for feather decomposition: alkaline hydrolysis and vapor pressure. However, this method destroyed essential amino acids such as methionine, lysine, and histidine, and was also very costly. This hydrolysis had low nutritional value (Matikeviciene et al., 2015; Adelere and Lateef, 2016). Keratin can be hydrolyzed by keratinase produced by some microorganisms, which naturally convert keratin into smaller, absorbable molecules (Bernal et al., 2006).

*In silico* analysis of the translated sequence of KRLr1 showed that this keratinase has a high aliphatic index, negative GRAVY value, and low Instability index, which are distinctive feature of stable and is non-polar bacterial proteins. An analysis of circular phylogenetic reveals a high amino acid similarity between the KRLr1 and other serine peptidases of the S8 family. The S8 family contains the serine endopeptidase subtilisin and its homologs, also known as the subtilase family, which are the second largest family of serine peptidases (Radisky et al., 2006; Di Cera, 2009). This family is divided into two subfamilies: S8A and S8B (Muszewska et al., 2011). The S8 family members have been observed in bacteria, archaea, and fungi and it has been reported that they are probably involved in nutrition. The S8 family has diverse functions, including acting as: (1) activation of lantibiotics and cytolysins, (2) catalyzation of the first step in the proteolytic activation of sterol regulatory element-binding protein, (3) role in the processing of antigenic peptides, and (4) the degradation of cholecystokinin (Rawlings and Barrett, 2004; Antalis et al., 2010). The enzymatic activity of KRLr1 was demonstrated by the identification of the conserved catalytic site consisting of repetitive Asp-His-Ser/Ans-His-Ser motifs in the active site cleft. Histidine amino acid plays the role of donor H and nucleophilic attack is carried out by serine amino acids in the presence of aspartic or/and asparagine amino acid (Di Cera, 2009).

The amino acid sequence analysis of PDB-BLAST in NCBI indicated that KRLr1 exhibited the highest structural homology with chain A of Subtilisin Carlsberg from *Bacillus licheniformis*, which is identified as serine peptidase and a member of the S8 family (Maynes et al., 2005). KRLr1 PDB modeling was performed using the crystallographic structure of Subtilisin Carlsberg at a 1.9-Å resolution as a template in MODELLER 9 V7. The structure was validated using ProSA after refinement. Z-Score was calculated to be −10.31, which demonstrated compatibility with the template. Furthermore, the Ramachandran plot related to homology modeling quality validation of the modeled 3D structure revealed that 98% of residues KRLr1 were classified as favored and allowed regions. The 3D model of the building indicated that the KRLr1 has three layers, with a seven-stranded β sheet sandwiched between two layers of helices, which is present in the known structure of the S8 family (Rawlings and Barrett, 1994). Furthermore, the motif of the nucleophilic elbow, containing the conserved Asp-Thr/Ser-Gly, His-Gly-Thr-His, and Gly-Thr-Ser-Met-Ala-Xaa-Pro (subfamily S8) located at the turn between β-strands and α-helixes, which this property could be considered as signatures specific to that category of the S8 family (Evans et al., 2000). This motif is known to facilitate easy access of the serine by the keratinase substrate. The topology of the active site of KRLr1 in the modeled structure has a strong match with the known template Subtilisin Carlsberg.

A virtual simulation from KRLr1 keratinolytic activity was designed based on the ability of KRLr1 in feather keratin degradation using docking and MD. Since in the experimental step, chicken feather keratin was used as a substrate for keratinase, in this study, we used the monomeric form of two β-keratins encoded on chromosome 2 and 25 chicken chromosomes to simulate keratinase-keratine docking and MD (Jin et al., 2017). Docking results approved a stronger interaction affinity KRLr1-FK4 complex than KRLr1-FK12. Involvation of serine, histidine, asparagine, and aspartate amino acids in the KRLr1-FK4 complex demonstrated that the KRLr1 active site has interacted with FK4 cleavage sites.

Simulation of docking results by MD showed the RMSD and RMSF emphasized that KRLr1 was more involved with FK4 protein than with the KRLr1-FK12 complex. The tendency for the RMSD and RMSF to increase in the KRLr1-FK4 complex compared to the KRLr1-FK12 complex may be due to the increased availability of amino acids in the active site of keratinase in the FK4 structure. Calculation of binding free energy by MM/PBSA analysis indicated that, although free energy plays a principal role in the formation of complexes of KRLr1-FK4 and KRLr1-FK12, van der Waals interaction energy and electrostatic interaction energy also play an significant role in the stability of these complexes. Furthermore, high levels of negative energy in the KRLr1-FK4 complex lead to the formation of more stable interactions between KRLr1-FK4 proteins compared to those in the KRLr1-FK12 complex.

In general, different keratin polypeptides are classified into different groups according to their unique physicochemical properties, molecular structure, and producing epithelial cells (Li, 2019). Keratinase are resistant to degradation by common proteases (pepsin, trypsin), however, the sequence and composition of their amino acids affect the fold, properties, function of the keratin fibers, and the rate of digestion by the keratins. Furthermore, the amino acid sequence also affects the secondary structure of keratins, which may be enriched in α-helix (typical for α-keratins) or β-sheet structures (typical for β-keratins) (Lange et al., 2016).

Although the mechanism of keratinase enzyme adsorption is not yet well understood, it is known that the higher the adsorption capacity, the greater the degree of keratin hydrolysis (Brandelli et al., 2010). After enzyme binding and cleavage of disulfide bonds, keratin changes its conformation and exposes multiple sites for the hydrolytic action of enzymes (Supuran et al., 2002). Most of the keratinases belong to large groups of serine proteases based on their mode of action. Serine proteases are a functionally rich and diverse group of proteases, with nucleophilic serine residues located in the enzyme’s active site (Vidmar and Vodovnik, 2018). The formation of a Serin-Histidine dyad in both the KRLr1-FK4 and KRLr1-FK12 complexes indicates that KRLr1 could interact with FK4 and FK12, although MD results demonstrated KRLr1-FK4 complexes was more active.

When employed as the substrate to compute the kinetic parameters, KRLr1 demonstrated the highest keratinolytic activity. KRLr1 demonstrated low K_m_ and high k_cat_/K_m_ in comparison to several other examined keratinase, showing a strong predilection for keratin, which caused the enzyme to hydrolyze peptide bonds more quickly than other assessed keratinases. The capacity of the enzyme to maintain activity at long term and the inborn structural stability of KRLr1 were demonstrated using thermodynamic correlations. By computing the thermodynamic parameters of the conversion of [S] and [E] to [ES], the enzyme’s E_a_, was determined. E_a_ is the quantity of energy required to initiate a reaction. The speed of the reaction increases with the lower energy of the molecules involved. ΔG shows the catalytic reaction’s spontaneity. ΔH and ΔS shows the transition state’s (TS) efficiency (Sharma et al., 2020; Shahi et al., 2021) In general, KRLr1 had lower values of E_a_^‡^, ΔG^‡^, and ΔG^‡^_E–T_ compared to ΔH^‡^, ΔS^‡^, and ΔG^‡^_E–S_ for the thermodynamic parameters of ES complex formation. These findings suggested that the KRLr1 reaction possesses an effective transition state and is probably quicker. The foundation for calculating the thermodynamic parameters of irreversible thermal inactivation is the acquisition of the k_in_, and t_1/2_. The protein transition state (T) computation can be used to identify parameters of the irreversible thermodynamic.

Proteins are thought to become irreversibly denaturated through a two-step reaction known as N ↔ U → I, where N stands for their native state, U for a partially unfolded or reversible state, and I for an irreversible or inactivated state (Mosallatpour et al., 2019). Calculating the activation energy required for an enzyme’s irreversible heat inactivation is a crucial step in comprehending an enzyme’s thermal capacity.

In the course of its irreversible thermal inactivation process, KRLr1 displayed significant E_a_^#^ and ΔG^#^ values. Thermodynamic analysis of other keratinase enzymes revealed a similar rise in ΔG^#^ in the transition state with rising temperatures (Table 3). In contrast, highest ΔH^#^ and ΔS^#^ at the favored activity temperature are correlated with the enzyme’s stability and resistance to the denaturation process. At rising temperatures, the fall of ΔH^#^ and ΔS^#^ shows that KRLr1 structure is shifting toward the transition state. Elevated levels of ΔG^#^ and E_a_^#^, however, reveal that the KRLr1 necessitates a significant quantity of thermal inactivation energy for denaturation, which is why the KRLr1 opposes the occurrence of transition states. For the enzyme keratinase, similar results in transition state values (E_a_^#^ and ΔG^#^) have been seen (Table 3). These results demonstrate KRLr1’s thermal resilience when it is in the TS phase. Thermodynamics findings suggest that KRLr1’s stability at the phase of TS probably is inherited from its catalytic effectiveness.

One of the prominent features of serine proteinase group keratinase is that they are inhibited by PMSF (Laurila, 2013). *Bacillus licheniformis* is known as a potential host for the production of efficient keratinolytic enzymes for industrial applications (Lin et al., 1992; Porres et al., 2002; Radha and Gunasekaran, 2007). The keratinase gene of *Bacillus licheniformis* KRLr1 was amplified using specific primers and after removing the peptide signal, its rare codons were optimized and synthesized in the expression vector pET-21b (+). Radha and Gunasekaran (2007) reported in their studies that the presence of the keratinase peptide signal would reduce the expression of the keratinase enzyme derived from *Bacillus licheniformis* MKU3 in *E. coli* BL21(DE3). These researchers observed significant expression in their study after deletion of the peptide signal, and all of the protein produced was observed as inclusion bodies, which was consistent with the results of our study. In our study, we observed high expression of the keratinase gene after removing the peptide signal, and the protein produced was observed as inclusion bodies. As a result, using the modified Qiagen handbook method, the expressed protein was removed from the inclusion bodies. In SDS-PAGE gel, a protein with a molecular weight of kilodaltons was observed. Western blot results confirmed the expression of recombinant keratinase.

In determining the properties of the recombinant enzyme in the temperature range of 15–80°C, it was found that the highest enzyme activity was at 37°C and the enzyme has a stable activity in the temperature range of 30–70°C. The optimum pH of enzyme activity was found at pH 6–8 and its highest activity is at pH 6. The results of our study are similar to the results of Hu et al. (2013) and those of *Bacillus licheniformis* S90 and other recombinant keratinases (Radha and Gunasekaran, 2007). Liu et al. (2013) reported that, after cloning and expression of keratinase gene derived from *Bacillus licheniformis* BBE11-1 in *Bacillus subtilis* WB600 under PHpaII promoter, its temperature and pH optimum were 40 and 10.5°C (Liu et al., 2013). They also determined that the enzyme is stable in the temperature range of 10–50°C and pH 7–11.5 (Liu et al., 2013). The optimum pH of keratinase activity of most bacteria, fungi and actinomycetes is in the range of neutral to alkaline, and the optimum temperature of their activity is in the range of 30–80°C (Pandey et al., 2019). Manonmani et al. (2015), reported in their study that keratinase enzyme obtained from *Bacillus tropicus* strain Gxun-17 has the highest activity at neutral pH close to alkaline. Rios et al. (2022) investigated the *Pedobacter* sp. 3.14.7 strain among the 7 bacterial strains with keratinase activity isolated from Antarctic birds’ nests. These researchers stated that the activity of the enzyme on the feather substrate is about 85%, and the optimal pH and temperature of its activity are 7.5 and 55°C, respectively (Rios et al., 2022).

In our study, we observed that 2-mercaptoethanol stimulates the activity of the enzyme, but PMSF and IAA inhibits its activity. These results were consistent with the results of Nnolim and Nwodo (2020) who stated that, among all reducing agents, 2-mercaptoethanol has a stimulating effect on keratinase activity. Also, Shen et al. (2022) in their studies investigating the effect of keratinase activity obtained from *B. tropicus* Gxun-17, reported that PMSF inhibits keratinase activity.

In examining the effect of metal ions on keratinase activity, it was found that Ca^2+^, Fe^2+^, and Mg^2+^ increase the activity of the enzyme. But by increasing the concentration of these ions from 5 to 10 mM, the activity of the enzyme decreases. These results were consistent with the results of Jana et al. (2022). These researchers reported that enzyme activity increases in the presence of Ca^2+^ and Mg^2+^. But Shen et al. (2022) stated that increasing the concentration of Na^2+^, Mg^2+^, and Ca^2+^ ions limits enzyme activity.

Among the organic solvents and surfactants used in this study, Tween 20 and glycerol increased the enzyme activity. But with the increase in the amount of these substances and the presence of SDS, the activity of the enzyme was gradually limited. The results of our study were similar to the results of Rios et al. (2022), these researchers showed in their study that Tween20 gradually inhibit the enzyme activity if it exceeds a certain amount, and the enzyme in the presence of SDS is inhibited by about 24.1% (Rios et al., 2022).

In this study, digestion of feathers was performed using recombinant keratinase. As a result of this study, digestible amino acids were analyzed using HPLC. The results of HPLC analysis showed that recombinant keratinase had high activity and amino acids obtained from the activity of this enzyme had admirable concentrations.

The content of the total amino acids obtained from feather digestion in this study was 854 mg/liter, and the amino acids cysteine, phenylalanine, tyrosine, and lysine had the highest amounts, compared to other amino acids, respectively. Phenylalanine and tyrosine are essential amino acids that cannot be produced by the body. Therefore, the resulting keratinase can play an important role in the poultry diet containing feather powder for the production of these amino acids. Peng et al. (2019) investigated the amino acids obtained from the digestion of feathers by the keratinase of co-cultivation *B. licheniformis* BBE11-1 and *S. maltophilia* BBE11-1 using the HPLC method and reported in their results that the total content of amino acids was 895.89 mg/L after 48 h of digestion. In the study of these researchers, the amino acids tyrosine, valine, phenylalanine and leucine had the highest amount, respectively. In a previous study by Fang et al. (2013) the effect of *Stenotrophomonas maltophilia* BBE11-1 on wool was investigated, and the amount of phenylalanine amino acid in this study was the highest among other amino acids.

Radha and Gunasekaran (2007) reported that the recombinant keratinase from *B. licheniformis* MKU3 was three times more active and produced more enzyme than the keratinase from the wild type strain *B. licheniformis* MKU3. Fisher et al. (1981) stated that the different combinations of feather amino acids varied depending on different processing methods, such as hydrolysis. These researchers also reported that differences in the amino acid composition of the feather may be due to the use of different parts of the feather as well as the different ages of the bird, particularly methionine, threonine, isoleucine, and valine, which vary with the age of the bird (Fisher et al., 1981). Keratinase wastes are rich in many of the amino acids necessary to enhance animal nutrition (Onifade et al., 1998). A study reported that addition of keratinase to the diet improved immune system responses, weight gain, digestibility, intestinal morphology, and the growth of young pigs (Café et al., 2002; Wang et al., 2011). Versazyme and Cibenza DP100TM are two keratinase-based dietary additives that are naturally produced as a fermentation product from *Bacillus licheniformis* PWD-1. Adding Versazyme to powdered and pelleted diets in poultry feed has a significant effect on the growth and feed intake of broilers (Stark et al., 2009). While Cibenza DP100TM is used to strengthen the immune system in the growth of young pigs (Wang et al., 2011).

## Concluding remarks and future prospect

Recombinant keratinase has been studied intensively and has been demonstrated to be an efficient enzyme for degrading keratin-rich waste streams. Its potential applications in the field of bioremediation, bioenergy production, and food industry have been studied extensively. It is expected that with further research and development in this field, KRLr1 recombinant keratinase will continue to be an important tool for the efficient and cost-effective degradation of keratin-rich waste streams. Furthermore, with the discovery of more efficient and specific keratinases, the use of recombinant keratinases may be extended to other industries such as the pharmaceutical, cosmetic, and medical fields. Therefore, the future outlook of KRLr1 looks promising and will continue to be of great importance in the future.

## Data availability statement

The datasets presented in this study can be found in online repositories. The names of the repository/repositories and accession number(s) can be found in the article/Supplementary material.

## Author contributions

SR, AM, and JF: conceptualization and funding acquisition. SR and AM: methodology. SR, JF, MB, MS, HB, HT, JZ, and YM: formal analysis. SR and AA: resources. SR and MS: data curation. SR: writing—original draft preparation. SR, AM, JF, HM, and MH: writing—review and editing. AM: project administration. All authors have read and agreed to the published version of the manuscript.

## Funding

This study was supported by the Iran National Science Foundation (INSF) for funding this work (grant no. 91002789).

## Conflict of interest

The authors declare that the research was conducted in the absence of any commercial or financial relationships that could be construed as a potential conflict of interest.

## Publisher’s note

All claims expressed in this article are solely those of the authors and do not necessarily represent those of their affiliated organizations, or those of the publisher, the editors and the reviewers. Any product that may be evaluated in this article, or claim that may be made by its manufacturer, is not guaranteed or endorsed by the publisher.

## Footnotes

^1^http://www.expasy.ch/tools/dna.html

^2^http://web.expasy.org/protparam

^3^http://www.sciencelauncher.com/oligocalc.html

^4^http://pfam.xfam.org/search

^5^http://MEROPS.sanger.ac.uk

^6^http://www.cbs.dtu.dk/services/NetSurfP/

^7^http://mordred.bioc.cam.ac.uk/~rapper/rampage.pHp

^8^https://zhanglab.ccmb.med.umich.edu/COFACTOR/

^9^https://www.cgl.ucsf.edu/chimera/

^10^https://www.expasy.org/resources/peptidecutter

^11^https://alcazar.science.uu.nl/services/HADDOCK2.2/haddockserver-easy.html

^12^https://cgenff.umaryland.edu/
