# Supplementary material for: Biochemical and molecular characterization of novel keratinolytic protease from Bacillus licheniformis (KRLr1)
Source: Front Microbiol. 2023 May 10;14:1132760. doi: 10.3389/fmicb.2023.1132760 (PMC10206251; doi:10.3389/fmicb.2023.1132760)
Supplement: Supplementary file 1 [file Data_Sheet_1.docx]

Graphical representation of HADDOCK results in Figure 8 has compared clusters of each KRL1-FK4 and KRL1-FK12 complexes based water refined model by HADDOCK. The clusters are calculated based on the interface-ligand RMSDs (i-l-RMSD) calculated by HADDOCK, with the interface defined automatically based on all observed contacts. HADDOCK score in comparison with i-l-RMSD showed an accumulation of cluster 1 points (red points) for both of LRRk1-FK4 (Fig 7A) and LRRk1-FK12 (Fig 7D) complexes. Further, reduce the scatter of points and RMSD [Å] in the graph of van der Wals vs i-RMSD (Fig 7B&E) and graph of Electrostatic vs i-RMSD (Fig 7C&F) regarding the LRRk1-FK4 and LRRk1-FK12 complexes, respectively, showed that cluster 1 of both complexes had most stable HADDOCK score.


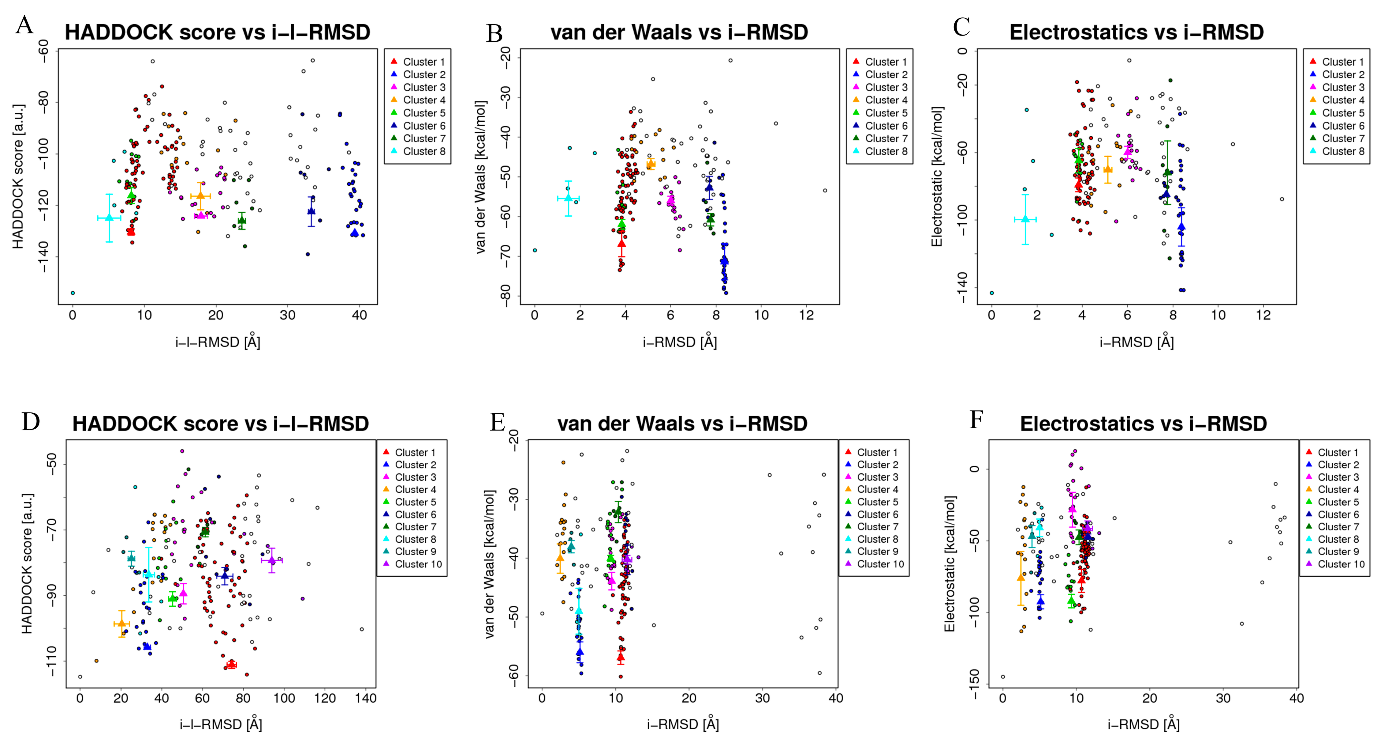


**Supplementary Figure S1.** **Graphical representation of HADDOCK results.** (A), (B) and (C) are referred to as HADDOCK score vs i-l-RMSD, van der Wals vs i-RMSD and Electrostatics vs i-RMSD of the KRL1-FK4 complex. In each graph, cluster 1 is shown in the red triangle. (D), (E) and (F) ) are referred to as HADDOCK score vs i-l-RMSD, van der Wals vs i-RMSD and Electrostatics vs i-RMSD of the KRL1-FK12 complex. In each graph, cluster 1 is shown in the red triangle.
